# Light‐Up Fluorescence and Circularly Polarized Luminescence in Achiral Interlocked Framework via Adaptive Lone Pair‐π Interaction Confinement

**DOI:** 10.1002/advs.202406890

**Published:** 2024-09-03

**Authors:** Yuan Wang, Xuefeng Zhu, Jianlei Han, Tongling Liang, Ningning Wu, Junfeng Xiang, Guanghui Ouyang, Minghua Liu

**Affiliations:** ^1^ Beijing National Laboratory of Molecular Sciences and CAS Key Laboratory of Colloid Interface and Thermodynamics Institute of Chemistry Chinese Academy of Sciences ZhongGuanCun North First Street 2 Beijing 100190 China; ^2^ Beijing National Laboratory for Molecular Science (BNLMS) Center for Physicochemical Analysis and Measurement Institute of Chemistry CAS ZhongGuanCun North First Street 2 Beijing 100190 China; ^3^ University of Chinese Academy of Sciences Beijing 100049 China

**Keywords:** adaptive chirality, circularly polarized luminescence, lp‐π interaction, metal–organic framework

## Abstract

Interactions between lone pairs and aromatic π systems are significant across biology and self‐assembled materials. Herein, employing an achiral confinement metal–organic framework (MOF) encapsulates guest molecules, it is successfully realized that lone pair (lp)‐π interaction induces fluorescence “turn‐on” and circularly polarized luminescence for the first time. The MOFs synthesized based on naphthalenediimide show nearly non‐emissive, which can be light‐up by introducing acetone or ester guests containing lone pairs‐π interaction. Furthermore, the introduction of a series of lp‐rich chiral esters induces supramolecular chirality as well as circularly polarized luminescence in achiral MOFs, while also observing chiral adaptability. This work first demonstrates the luminescence and chiral induction via lone pair electrons‐π interactions, presenting a fresh paradigm for the advancement of chiroptical materials.

## Graphical abstract

The first example of fluorescence light‐up and circularly polarized luminescence achieved by encapsulating acetone or ester guest molecules in achiral‐constrained MOFs through lone pair‐π interactions is reported. Introducing lone pair‐rich chiral esters further induces supramolecular chirality and circularly polarized luminescence, showcasing chiral adaptability.

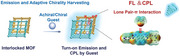

## 1 Introduction

Non‐covalent interactions play a crucial role in the formation of complex structures in biological system and the precise control of materials.^[^ ^1^ ^]^ Among various non‐covalent interactions, the π‐system involved non‐covalent interactions such as π–π,^[^ ^2^ ^]^ XH‐π,^[^ ^3^ ^]^ cation‐π,^[^ ^4^ ^]^ anion‐π,^[^ ^5^ ^]^ and lone‐pair (lp)‐π interactions^[^ ^6^ ^]^ are gaining increasing attention for their role in stabilizing higher‐order structures and the development of novel functional materials. Compared with the other π‐involved interactions, the significance of lp‐π interactions was demonstrated and recognized only recently.^[^ ^7^ ^]^ The lp‐π interactions not only affect the stability and reactivity of molecules but also play key roles in catalysis,^[^ ^1c^ ^]^ molecular recognition,^[^ ^8^ ^]^ adsorption,^[^ ^9^ ^]^ and the design of new materials. However, their application in the field of luminescent materials has been limited due to weak forces and difficulties in facilitating intermolecular charge transfer.^[^ ^10^ ^]^ It still remain a great challenge to develop luminescent materials based on the lp‐π interactions.

Metal–organic frames (MOFs), with ordered confined pores and flexible, adjustable structures, have been widely studied.^[^ ^11^ ^]^ They serve as effective platforms for studying weak interactions and chirality.^[^ ^11^, ^12^ ^]^ Recently, MOF materials with circularly polarized luminescence (CPL) have received much attention due to their dexterity in fabrication and wide range of interactions with guest molecules.^[^ ^11^, ^13^ ^]^ In general, to construct chiral MOF materials, chiral elements or scaffolds with the point or axis‐chirality need to be introduced into the framework of MOF.^[^ ^11^, ^14^ ^]^ However, the cumbersome synthesis and expensive chiral ligands have limited the development of chiroptical MOFs. Therefore, it is necessary to explore new mechanisms of action to expand chiroptical active MOF materials. It is possible to endow MOF chirality through the interplay with the chiral guest.^[^ ^14^, ^15^ ^]^ Here, we report the first example of the lighting‐up of achiral MOF into luminescent and CPL active materials through the nano‐confinement of the solvent via the lone‐pair‐π interaction.

**Figure 1:**
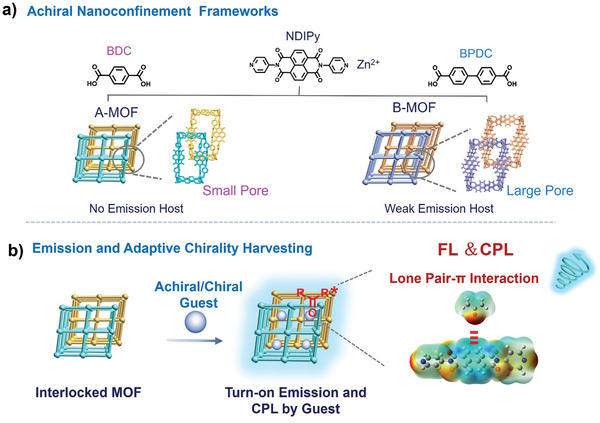
a) Construction of achiral nanoconfinement metal–organic frameworks (MOFs) with varying pore sizes. b) In achiral nanoconfinement MOFs, simultaneous luminescence and chirality harvesting by lp‐π interactions.

A series of 3D confined MOFs with varying dimensions were constructed based on dipyridine‐naphthalenediimide and aromatic dicarboxylic acids, as shown in **Figure** **1**a. By encapsulating acetone or ester guest molecules, we find that these MOFs could be lighted up with fluorescence. A lp‐π interaction in the confined cavity is suggested to induce the luminescence of MOF. By utilizing lp‐π interactions to encapsulate chiral ester guest molecules, we further achieved chirality transfer from guest to the achiral MOF and thus led to circularly polarized luminescence (Figure 1b). Furthermore, we found an adaptive effect of the solvent on the MOF cavity. Circularly polarized luminescence can be achieved only when the size of the MOF cavity can adapt the solvent. This work reveals the role of the confined lp‐π interaction in lighting up the luminescence as well as CPL in MOF materials and provides new insights for the development of chiroptical materials.

## 2 Results and Discussion

### 2.1 Synthesis and Characterization of Confined MOF Materials

A series of flexible, confined NDIPy (*N, N*′‐di(4‐pyridyl)‐1,4,5,8‐naphthalenediimide)‐based metal–organic frameworks were synthesized according to previous methods.^[^ ^16^ ^]^ NDI (1,4,5,8‐naphthalenediimide), with its electron‐deficient core, excels as an electrophilic pillar.^[^ ^11^, ^17^ ^]^ Luminescence via lone pair‐π interactions in confined spaces is yet unreported. Combining pillars (NDIPy) with co‐ligands of varying sizes yields 3D interpenetrating topological frameworks of different dimensions.^[^ ^11a^ ^]^ Thus,1,4‐dicarboxybenzene acid (BDC) and 4,4'‐biphenyldicarboxylic acid (BPDC) are effective in modulating the cavity sizes within MOFs. The MOFs constructed with 1,4‐dicarboxybenzene acid, named A‐MOF, exhibit small pore sizes. As depicted in Figure S1a (Supporting Information), within the stereo 3D topological structure of A‐MOF, interactions among BDC, Zn, and NDIPy create an effective nanoscale confined cavity (l ≈ 11.4–11.9 Å; h ≈ 6.7–7.2 Å), capable of constraining guest molecules. In contrast, assembled with BPDC, named as B‐MOFs, feature larger pore sizes. The single‐crystal structure of B‐MOF, as seen in Figures S1b–d (Supporting Information), showcases biphenyl‐4,4'‐dicarboxylic acid forming larger cubic topological 3D nanocavities (l ≈ 15.1–15.2 Å; h ≈ 10.4–11.4 Å). Adjacent and above the NDI group, larger nanocavities exist, capable of encapsulating guest molecules such as DMF, acetone, *t*‐butyl acetate, or larger‐sized guests. In these interpenetrating 3D topological frameworks, there are multiple cavities and active sites. However, the NDIPy as a π‐conjugated group, the face‐to‐face active sites with electron donor guest molecules, allow for efficient orbital overlap, providing a higher contribution in the excited state.^[^ ^18^ ^]^ Therefore, we focus our investigation on the interactions between active sites within the nanocavities on the π‐plane of DNIPy and guest molecules.

### 2.2 Lighting Up Luminescence of MOFs

**Figure 2:**
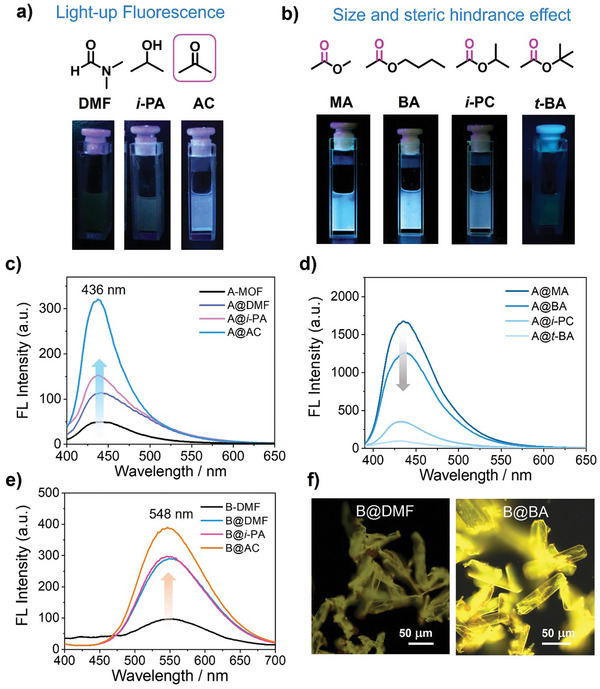
Fluorescence images of A‐MOF with encapsulated solvent guest molecules: a) lp‐π interaction induced fluorescence “turn on” and b) size and steric effects. Fluorescence spectra of A‐MOF with c) encapsulated *N, N*‐dimethylformamide, isopropanol, acetone, and d) methyl acetate, n‐butyl acetate, isopropyl acetate, t‐butyl acetate, *λ*_ex_ = 360 nm. Fluorescence spectra of e) B‐MOF with encapsulated *N, N*‐dimethylformamide, isopropanol, acetone. f) Fluorescence microscopy images of B‐MOF@DMF and B‐MOF@BA.

The original A‐MOF crystals formed in DMF are characterized by very low quantum yields and shorter lifetimes (**Figure** **2**a), considered to be in a luminescent “off” state.^[^ ^16^ ^]^ Removing inherent DMF from A‐MOF creates effective nanocavities while preserving luminescence silence. Intriguingly, A‐MOF encapsulating isopropanol guest molecules emit very weak fluorescence (as seen in Figure 2a,c), whereas when acetone guests are encapsulated within its nanoscale cavities, emit bright blue fluorescence at 436 nm, the fluorescence lifetime is 0.95 ns. We surmise this may be associated with the electron‐donating capability of carbonyl oxygen,^[^ ^19^ ^]^ as indicated by the electrostatic potential in Figure S6 (Supporting Information). Subsequently, encapsulating ester guest molecules containing carbonyl oxygen within A‐MOF, we similarly observe a bright blue fluorescence (Figure 2b; Figure S2, Supporting Information). The UV spectrum reveals absorption between 300–400 nm, indicative of the characteristic absorption peaks of NDI moieties (Figure S3, Supporting Information).^[^ ^20^ ^]^ As the size of the ester molecules increases, the fluorescence of A‐MOF gradually weakens (Figure 2d; Figures S4 and S5, Supporting Information). The electrostatic potential of the guest molecule reveals the distribution state of electrons and the sites of action, with the increasing steric hindrance of the ester side chains, potentially greatly impeding the interaction between the lone pair on the ester carbonyl oxygen and NDIPy (Figure S7, Supporting Information). The PXRD patterns of A‐MOF encapsulating ester guest molecules (MA, BA, *i*‐PC) exhibit similarities to the crystalline forms encapsulating DMF and acetone (Figure S8a–c, Supporting Information). The PXRD of A@*t*‐BA, with relatively large steric hindrance, also shows a certain degree of shift compared to the PXRD of the unencapsulated guest (Figure S8c, Supporting Information), indicating that A‐MOF can partially encapsulate tert‐butyl acetate guests. Therefore, the gradual weakening of A‐MOF fluorescence can be attributed to the combined effects of cavity size matching and steric hindrance between A‐MOF and the guest molecules. Unlike A‐MOF, B‐MOF inherently emits a faint yellow light at 548 nm. Similarly, fluorescence images and spectroscopy reveal that B‐MOF encapsulating carbonyl oxygen‐containing guest molecules exhibit enhanced fluorescence at 548 nm (Figure 2e,f; Figures S9 and S10, Supporting Information). Single crystal and PXRD results indicate that (Figure S11, Supporting Information), B‐MOF can also accommodate larger guest molecules due to its larger nanocavities.

### 2.3 Lone Pair‐π Interaction Mechanism in Confined MOFs

**Figure 3:**
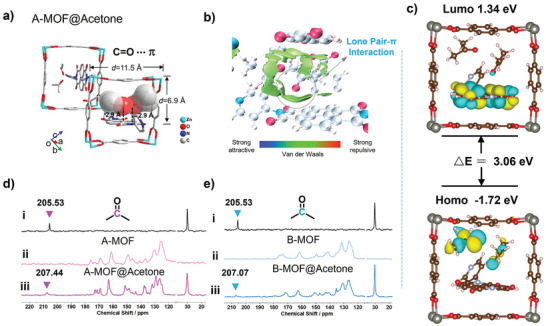
a) The crystal structure of A‐MOF@Acetone (CCDC: 2277070). b) IGMH analysis of the optimized structure of A‐MOF@Acetone. Green isosurfaces show weak noncovalent interactions. c) The HOMO (Highest Occupied Molecular Orbital) and LUMO (Lowest Unoccupied Molecular Orbital) energy levels of A‐MOF@Acetone. ^13^C CP‐MAS NMR spectra of d) A‐MOF@Acetone and e) B‐MOF@Acetone, respectively. i) acetone solvent; ii) A‐MOF solventless and B‐MOF solventless; iii) A‐MOF@Acetone (pink line) and B‐MOF@Acetone (blue line).

To explore the aforementioned luminescence mechanism. Single crystal structure serves as a robust means to analyze the interaction mechanisms between host and guest molecules. Fortunately, we have successfully obtained the single crystal structure of A‐MOF@Acetone (**Figure** **3**a). Similar to the original A@DMF, the space above NDIPy can accommodate two guest molecules (Figure S1a, Supporting Information). The crystal structure clearly reveals that acetone molecules are confined within nanocavities and anchored through multiple non‐covalent interactions. There is a C═ O… π interaction (C═O… π = 2.9 Å) between the electron‐rich carbonyl oxygen and the electron‐deficient NDI π‐system. This demonstrates the presence of lone pair π‐interaction forces.

Utilizing theoretical calculation to elucidate the interactions between lp‐π in nanoconfinement. The structure of the single crystal was optimized, and non‐covalent interactions (NCI) were analyzed using the Independent Gradient Model based on Hirshfeld (IGMH),^[^ ^21^ ^]^ as detailed in the supplementary information. Figure 3b demonstrates that acetone molecules have non‐covalent interactions with the confined spaces within the MOF, with a notable lone pair‐π interaction between the carbonyl oxygen and the NDI group (Figure S12, Supporting Information). The Highest Occupied Molecular Orbital (HOMO) and the Lowest Unoccupied Molecular Orbital (LUMO) are critical for assessing the excited state characteristics of molecules and their capacity for interactions. Therefore, we conducted an analysis of the HOMO and LUMO energy levels within the crystal structure to uncover the impact of lp‐π interactions on the electronic attributes of molecules. As seen in Figure 3c, within the confined framework cavities, HOMO electron distribution is found on the acetone molecule, while the lowest unoccupied molecular orbital's electron cloud is primarily located on the NDI moiety. This indicates that in the excited state, electron‐rich acetone can transfer electrons to the electron‐deficient NDIPy inducing luminescence. Notably, after removing the crystal framework and independently calculating the energy levels of NDIPy and acetone molecules (Figure S13, Supporting Information), the results indicated that the HOMO and LUMO levels are predominantly localized on the NDIPy moiety. This demonstrates that confined cavities play a pivotal role in the lp‐π interaction‐induced luminescence.

The ^13^C CP‐MAS NMR (Figure 3d,e) results indicated a downfield shift of the carbonyl peak for acetone solvent in A‐MOF and B‐MOF from 205.53 ppm to 207.44 ppm (downfield shifts of 1.91 ppm) and to 207.07 ppm (downfield shifts of 1.54 ppm), respectively. This shift is attributed to the lp‐π interaction between the carbonyl oxygen within the confined nanocavities and the electron‐deficient NDI, reducing the electron density around the carbonyl carbon and diminishing the nuclear shielding effect. In conclusion, through experimental and theoretical calculations, we elucidated the mechanism of luminescence induced by lp‐π interactions within nanoscale confined cavities.

### 2.4 Adaptability and Chiroptical Properties in Achiral MOFs

**Figure 4:**
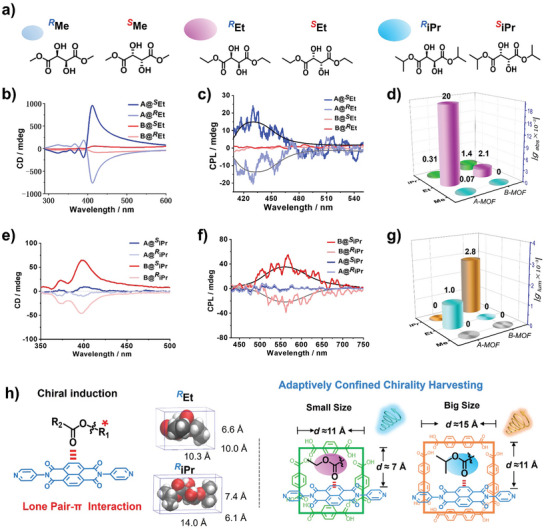
a) Schematic representation of the tartrate chiral guests molecule structures of *^R/S^* Me, *^R/S^* Et, and *^R/S^* iPr. CD spectra of b) MOF@*^R/S^* Et and e) MOF@*^R/S^* iPr. CPL spectra of c) MOF@*^R/S^* Et and f) MOF@*^R/S^* iPr. Comparison of *g*_abs_ and *g*_lum_ of the d) MOF@*^R/S^* Et and g) MOF*^R/S^* iPr. h) Schematic illustration of the mechanism of chiroptical induced by lp‐π interaction between chiral guest molecules and nanoconfinement MOFs. (Dimethyl tartrate is abbreviated as Me, diethyl tartrate is abbreviated as Et, and diisopropyl tartrat is abbreviated as iPr).

We proceeded to encapsulate a variety of chiral solvent guest molecules, each featuring carbonyl oxygen, into achiral‐constrained MOFs to study their chiroptical properties. We have chosen chiral guests of various sizes that can turn on emission: *R/S*‐methyl lactate (*^R/S^* Lac), dimethyl‐*R/S*‐tartrate (*^R/S^* Me), diethyl‐*R/S*‐tartrate (*^R/S^* Et), diisopropyl‐*R/S*‐tartrate (*^R/S^* iPr), dimethyl 2,3‐o‐isopropylidene‐*R/S*‐tartrate (*^R/S^* isoT) (**Figure** **4**a; Figures S14–S17, Supporting Information). Initially, we selected smaller chiral guest molecules, *^R/S^* Lac and *^R/S^* Me, for encapsulation into A‐MOF, where we detected weak CD signals, with |*g*_abs_| values of 6.3 × 10^−5^ and 7 × 10^−5^, respectively (Figure 4d; Figures S18a,  S19a and Table S1, Supporting Information). No CPL signals were detected in A‐MOF (Figure 4g; Figures S18c and S19c, Supporting Information), possibly due to the guest molecules not being adequately fixed within the confined cavities, leading to an excited state chirality too weak to be detected by the instrument. Further, increasing the size of the guest molecules, as illustrated in Figure 4b, A@*^R^* Et manifested an exceptionally intense Cotton effect at the NDI UV absorption peak with a |*g*_abs_| value reaching as high as 0.02 (Figure 4b,d). A@*^S^* Et presented a mirror‐symmetrical CD signal. Due to lp‐π interactions facilitating luminescence and simultaneously capturing chirality, A@*^R^* Et displayed a negative CPL signal at the fluorescence emission peak (Figure 4c), delineating the chiral features in the excited state. A@*^S^* Et revealed a mirror‐symmetrical CPL signal, with a |*g*_lum_| value of 1 × 10^−3^ (Figure 4g). The dissymmetry factor (*g*_lum_ = 2(*I*_L_ – *I*_R_)/(*I*_L_ + *I*_R_), where *I*_L_ and *I*_R_ are the intensities of left and right circularly polarized light, respectively) can be determined to quantify the efficiency of chirality induction in this process.^[^ ^22^ ^]^ By rotating and inverting the samples, we assessed the impact of linear dichroism (LD) on the circular dichroism (CD) signals, clarifying that its influence on CD intensity is negligible (Figure S20a, Supporting Information). Furthermore, the effect of linear birefringence in MOF sample suspensions on the circularly polarized light (CPL) signal was investigated, indicating that its impact on CPL intensity is negligible (Figure S20b, Supporting Information). Additionally, when pure NDIPy powder was mixed with *^R^* Et for chiral induction (Figure S21, Supporting Information), no CD signal was generated. This underscores that effectively confined cavities are crucial for the generation of chiral signals. However, encapsulating the larger‐sized *^R/S^* iPr chiral solvent and the rigid *^R/S^* isoT chiral solvent into A‐MOF respectively resulted in CD signals with a |*g*_abs_| value of 0.31 × 10^−3^ (Figure 4e,g) and |*g*_abs_| = 1.4 × 10^−3^ (Figure S22a and Table S2, Supporting Information), both significantly smaller than that of A@*^R/S^* Et (|*g*_abs_| = 0.02).

The CPL signals for both A@*^R/S^* iPr and A@*^R/S^* isoT also remained silent (Figure 4f,g; Figure S22c and Table S2, Supporting Information). We speculate that this may be due to the smaller size of A‐MOF confined cavities being poorly adapted to accommodate larger or rigid chiral guest molecules.

Next, we switched to the larger‐sized B‐MOF. B‐MOF encapsulating *^R/S^* Lac and *^R/S^* Me exhibited silence in both CD and CPL signals (Figures S18b,d,  S19b,d, and Table S1, Supporting Information), attributing to the larger confined cavities. Subsequently, B@*^R/S^* Et exhibited a CD signal with a |*g*_abs_| value of 2.1 × 10^−3^ (Figure 4b), which is relatively weaker compared to A@*^R/S^* Et, while the CPL signal remained silent (Figure 4d). Contrarily, when the large‐sized *^R/S^* iPr chiral solvent induced a CD signal in B‐MOF with a |*g*_abs_| value of 1.4 × 10^−3^, which is higher in intensity compared to A@*^R/S^* iPr (|*g*_abs_| = 0.31 × 10^−3^) (Figures 4e,d). B@*^R/S^* iPr exhibited a mirror‐symmetric CPL at the emission peak, with a |*g*_lum_| value of 2.8 × 10^−3^ (Figures 4f,g). This is due to the larger‐sized B‐MOF having better adaptability to large‐sized chiral guest molecules. For more rigid chiral molecules *^R/S^* isoT, B@*^R/S^* isoT exhibited CD signals (Figure S22b and Table S2, Supporting Information), with a |*g*_abs_| value of 3.3 × 10^−3^ being higher than A@*^R/S^* isoT (|*g*_abs_| = 1.4 × 10^−3^), due to B‐MOF possessing suitably confined cavities. B@*^R/S^* isoT exhibited a CPL signal with |*g*_lum_| = 3.6 × 10^−3^ (Figure S22d and Table S2, Supporting Information), suggesting the presence of a favorable size‐matching effect within B‐MOF. XRD analysis indicates that both A‐MOF and B‐MOF show shifts upon encapsulating *^R^* Et and *^R^* iPr guest molecules, a manifestation of the framework's adaptability in accommodating guest molecules (Figure S23, Supporting Information). The above experimental results corroborate our hypothesis that B‐MOF, with its large confined cavities, exhibits better adaptability to large‐sized chiral guests and rigid molecules (Figure 4h).

To further elucidate the impact of the adaptive characteristics, we synthesized C‐MOF (Figure S24, Supporting Information), whose crystallographic topology revealed that the NDIPy core is tightly encapsulated by biphenyl dicarboxylate linkers, lacking effective interaction cavities (Figure S25, Supporting Information). Furthermore, XRD analysis indicated that there was no significant shift consistent after soaking with *^R^* Et and *^R^* iPr guest molecules, suggesting that the chiral guest molecules did not infiltrate the framework, and the framework lacks adaptability (Figure S26, Supporting Information). Concurrently, C‐MOF exhibited chiroptical silence in the chiral solvents (Figures S27 and S28, Supporting Information). The aforementioned data indicate that effective interaction sites and the characteristics of an adaptive framework are crucial for the induction of chirality.

## 3 Conclusion

In summary, we utilized 3D nanoconfined MOFs to encapsulate guest molecules with lp‐π interaction and lighting up the MOFs with fluorescence as well as circularly polarized luminescence (CPL). The MOFs synthesized from *N, N*‐di(4‐pyridyl)‐1,4,5,8‐naphthalenediimide (NDIPy) and 1,4‐dicarboxybenzene acid (BDC) or 4,4'‐biphenyldicarboxylic acid (BPDC) showed almost no/weak emissive. However, upon dispersing into acetone or ester solvents, luminescence was lighted up for the MOF. It was revealed that the novel lp‐π interaction between the carbonyl of ketone or ester with the π‐conjugated system of the MOF in the confined cavity is mainly responsible for such luminescence. Furthermore, by incorporating lp‐rich chiral esters into MOFs, chirality can be induced in MOFs thus leading to CPL. A size match between the MOF cavity and chiral ester is also revealed. This work not only uncovers the role of weak lp‐π interactions in lighting up the luminescence of MOFs but also opens new avenues for the design and fabrication of novel optically active materials.

## 4 Experimental Section

### Materials

All the chemical substances were utilized as obtained, with no additional purification. *N, N*′‐di(4‐pyridyl)‐1,4,5,8‐naphthalenediimide, 1,4‐dicarboxybenzene, 4,4'‐biphenyldicarboxylic acid, zinc nitrate hexahydrate, dimethyl‐*R/S*‐tartrate, diethyl‐*R/S*‐tartrate, diisopropyl‐*R/S*‐tartrate, *S/R*‐methyl lactate, dimethyl 2,3‐o‐isopropylidene‐*R/S*‐tartrate were purchased from TCI. *N, N*′‐dimethylformamide, dichloromethane, hexane, acetone, isopropyl alcohol, methyl acetate, n‐butyl acetate, isopropyl acetate, tert‐butyl acetate were purchased from J&K.

### Instruments and Characterization

UV‐vis spectra were obtained through JASCO UV‐550 and Hitachi UV‐3900 spectrometers. Samples were conditioned for examination in quartz cuvettes with light paths of 0.1 and 1 mm. The fluorescence spectra were recorded on a Hitachi F‐4600 fluorescence spectrophotometer. The samples were prepared for testing on quartz cuvettes with a light path of 0.1 mm and 1mm, the testing voltage was 400 V, and the width of the excitation and emission slits was 4 nm. Evaluated using the absolute method with a FLS980 instrument, utilizing an integrating sphere. The sample MOF@guest molecule was placed in a quartz slide for testing. CD spectra were recorded using JASCO 1500 and JASCO 1700 spectrophotometers. Samples were readied for analysis in quartz cuvettes with a 1 mm light path. The electronic circular dichroism (ECD) and linear dichroism (LD) spectra were documented, with LD contributions to CD being minimal and negligible in the experimental findings. CPL spectra were acquired using JASCO CPL‐200 and JASCO CPL‐300 spectrophotometers. The sample was readied by depositing a droplet of suspensions into a quartz cuvette with a 1 mm light path. Linear birefringence (LB) effects and potential artifacts from macroscopic anisotropy were largely mitigated by altering and adjusting the sample angle along the incident light propagation direction. ^13^C CP‐MAS NMR spectra were recorded on a Bruker ADVANCE III 400 spectrometer at 298 K and 258k. Fluorescence microscope images were obtained on olympus IX83. A small amount of the sample was placed on a glass slide for observation. SXRD data were gathered using an XtaLAB Synergy‐R diffractometer. The structures were deciphered via direct methods and honed through a full matrix least squares approach based on F2, utilizing the SHELXL 97 program (Sheldrick, 1997). The crystal drawing were obtained using the software Mercury and Diamond.

### Theoretical Calculation

Detailed information is provided in the theoretical calculation in the Supporting Information.

### Synthesis of the MOFs

All MOFs were synthesized, and MOFs encapsulate guest molecules detailed information is provided in the materials and methods in the Supporting Information.

[A‐MOF@Acetone (CCDC: 2277070), A‐MOF@DMF (CCDC: 2340866), B‐MOF@DMF (CCDC: 2340869), B‐MOF@Acetone (CCDC: 2340868), B‐MOF@*t*‐BA (CCDC: 2340870), C‐MOF@DMF (CCDC: 2340871) contains the supplementary crystallographic data for this paper. These data can be obtained free of charge from The Cambridge Crystallographic Data Centre via www.ccdc.cam.ac.uk/data_request/cif.]

## Conflict of Interest

The authors declare no conflict of interest.

## Supporting information

Supporting Information

## Data Availability

The data that support the findings of this study are available in the supplementary material of this article.
