# Supplementary material for: Light‐Up Fluorescence and Circularly Polarized Luminescence in Achiral Interlocked Framework via Adaptive Lone Pair‐π Interaction Confinement
Source: Adv Sci (Weinh). 2024 Sep 3;11(40):2406890. doi: 10.1002/advs.202406890 (PMC11516062; doi:10.1002/advs.202406890)
Supplement: Supplementary file 1 — Supporting Information [file ADVS-11-2406890-s001.docx]

**Supplementary Information for**

**Light-Up Fluorescence and Circularly Polarized Luminescence in Achiral Interlocked Framework via Adaptive Lone Pair-π Interaction Confinement**

*Yuan Wang, ^1^ Xuefeng Zhu,^1^ Jianlei Han, ^1^ Tongling Liang, ^2^ Ningning Wu, ^2^ Junfeng Xiang, ^2^ Guanghui Ouyang^1^ and Minghua Liu*^1,3^*

^1^ Beijing National Laboratory of Molecular Sciences and CAS Key Laboratory of Colloid, Interface and Thermodynamics, Institute of Chemistry, Chinese Academy of Sciences, ZhongGuanCun North First Street 2, Beijing 100190, China.

^2^ Beijing National Laboratory for Molecular Science (BNLMS), Center for Physicochemical Analysis and Measurement, Institute of Chemistry, CAS, ZhongGuanCun North First Street 2, Beijing, 100190, China.

^3^ University of Chinese Academy of Sciences, Beijing, 100049, China.

*Correspondence to: liumh@iccas.ac.cn

Contents

1. Experimental 3

1.1 Theoretical calculation 3

2. The synthesis of Metal-Organic Frameworks (MOFs) 3

2.1 MOFs Encapsulate Guest Molecule 4

3. The properties of MOFs encapsulated guest molecules 5

4. Adaptive chirality in achiral nanoconfined MOFs 14

5. Single crystal data 24

6. References 30

# 1. Experimental

# 1.1 Theoretical calculation

The guest molecules structure optimization calculated by Density Functional Theory (DFT), Gaussian 16^1^ Package, utilizing the B3LYP^2^ -D3(BJ)^3^ /6-311G(d,p) level.

The geometry optimizations and electronic structural analyses of A-MOF@Acetone were conducted via Density Functional Theory (DFT) employing the CP2K^4^ (version 2023.1), utilizing the PBE0^5^-D3(BJ)-ADMM^6^/DZVP-MOLOPT-SR-GTH level of theory. Additionally, an independent gradient model based on Hirshfeld (IGMH) was used to analyze noncovalent interactions (NCI)^7^

The distribution of electrons and holes during electron excitation was analyzed using Multiwfn3.8^8-9^(dev). The structure used in the calculations was obtained from the crystallographic data.

The visualization of the results was achieved with the aid of the tools VMD^10^ and VESTA^11^.

2. The synthesis of Metal-Organic Frameworks (MOFs)

The synthesis of A-MOF: *N, N*’-di(4-pyridyl)- 1,4,5,8-naphthalenediimide (0.21 g, 0.5 mmol), 1,4-dicarboxybenzene acid (0.083 g, 0.5 mmol), Zinc nitrate hexahydrate (0.149 g, 0.5 mmol) were dispersed in *N, N*’-dimethylformamide (50 mL) in a within the reaction kettle, and following ultrasonic mixing for uniformity, the mixture was heated to 95°C. After a reaction period of 48 hours, it was cooled down to room temperature to obtain MOF crystals.

The synthesis of B-MOF: *N, N*’-di(4-pyridyl)- 1,4,5,8-naphthalenediimide (0.105 g, 0.25 mmol), 4,4'-biphenyldicarboxylic acid (0.121 g, 0.5 mmol), zinc nitrate hexahydrate (0.149 g, 0.5 mmol) were dispersed in *N, N*’-dimethylformamide (50 mL) in a within the reaction kettle, and following ultrasonic mixing for uniformity, the mixture was heated to 80°C. After a reaction period of 48 hours, it was cooled down to room temperature to obtain MOF crystals.

The synthesis of C-MOF: *N, N*’-di(4-pyridyl)- 1,4,5,8-naphthalenediimide (0.21 g, 0.5 mmol), 4,4'-biphenyldicarboxylic acid (0.121 g, 0.5 mmol), zinc nitrate hexahydrate (0.149 g, 0.5 mmol) were dispersed in *N, N*’-dimethylformamide (50 mL) in a within the reaction kettle, and following ultrasonic mixing for uniformity, the mixture was heated to 95 °C. After a reaction period of 48 hours, it was cooled down to room temperature to obtain MOF crystals.

# 2.1 MOFs Encapsulate Guest Molecule

In the original solution containing MOF, dichloromethane was added and allowed to soak for 60 minutes, followed by the evaporation of the solvent, a process repeated thrice. Subsequently, n-hexane was introduced for guest exchange, the solvent was again evaporated, and this procedure was reiterated three times, resulting in solvent-free MOF. The aforementioned MOF material was directly immersed in the solvent of the respective guest molecules, with fresh solvent being replaced multiple times during the process. The process can be expedited through appropriate heating or by grinding, enabling the attainment of MOF material encapsulating the corresponding guest.

# The properties of MOFs encapsulated guest molecules


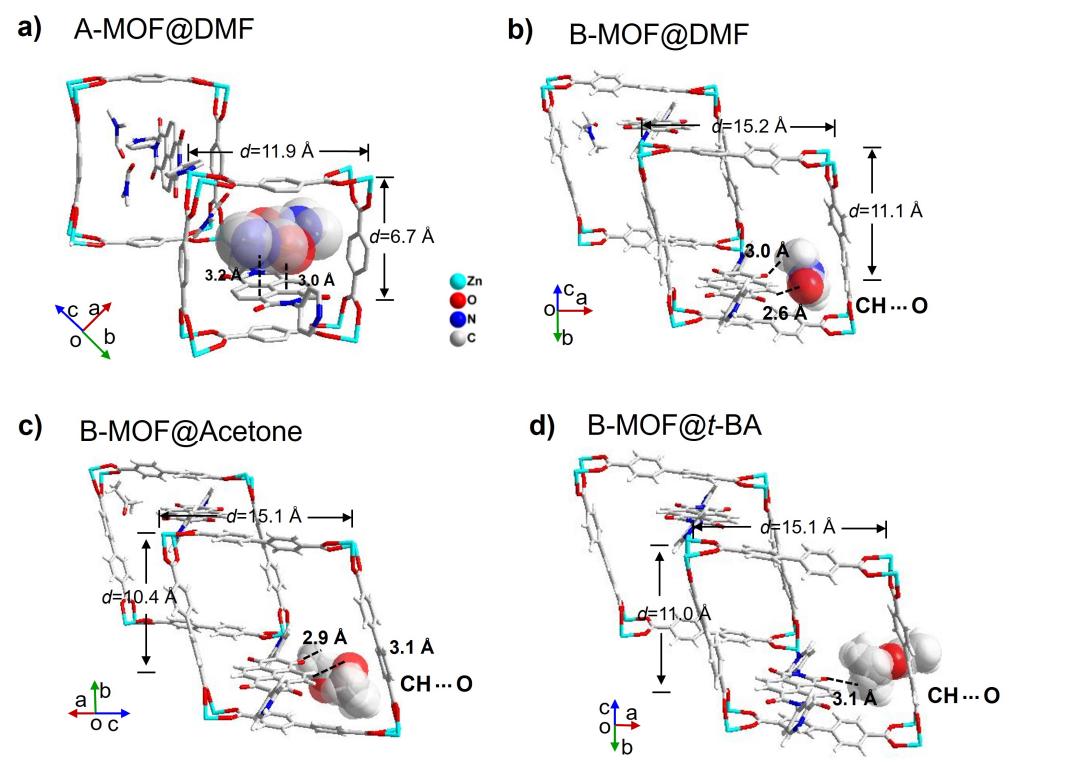


**Figure S1.** The crystal structure of A-MOF@DMF (CCDC: 2340866) (a), B-MOF@DMF (CCDC: 2340869) (b), B-MOF@Acetone (CCDC: 2340868) (c) and B-MOF@*t*-BA (CCDC: 2340870) (d).


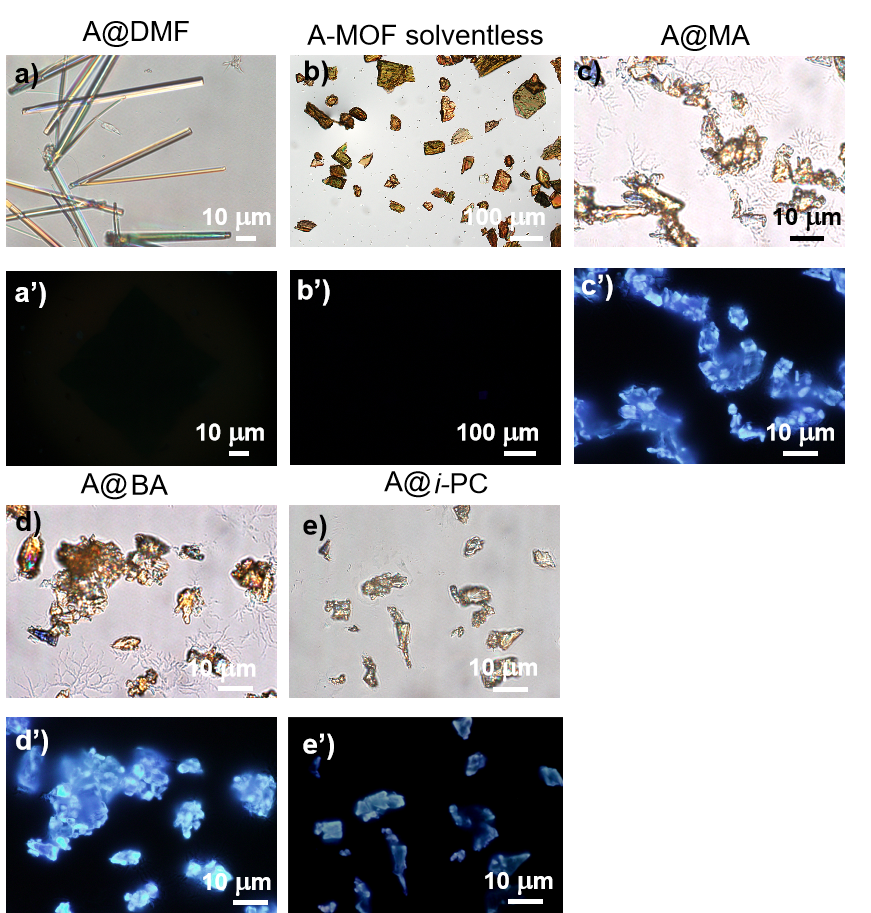


**Figure S2.** Fluorescence microscopy images: (a, a’) A-MOF@DMF; (b, b’) A-MOF solventless; (c, c’) A-MOF@EA; (d, d’) A-MOF@BA; (e, e’) A-MOF@*i*-PC (a-e) in the natural light, (a’-e’) excited by DAPI (361-389 nm).


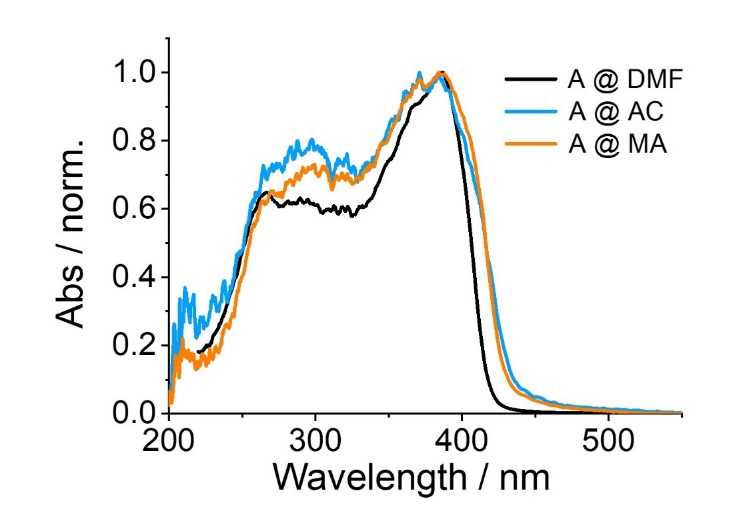


**Figure S3.** UV-Vis spectra of A-MOF@guests.


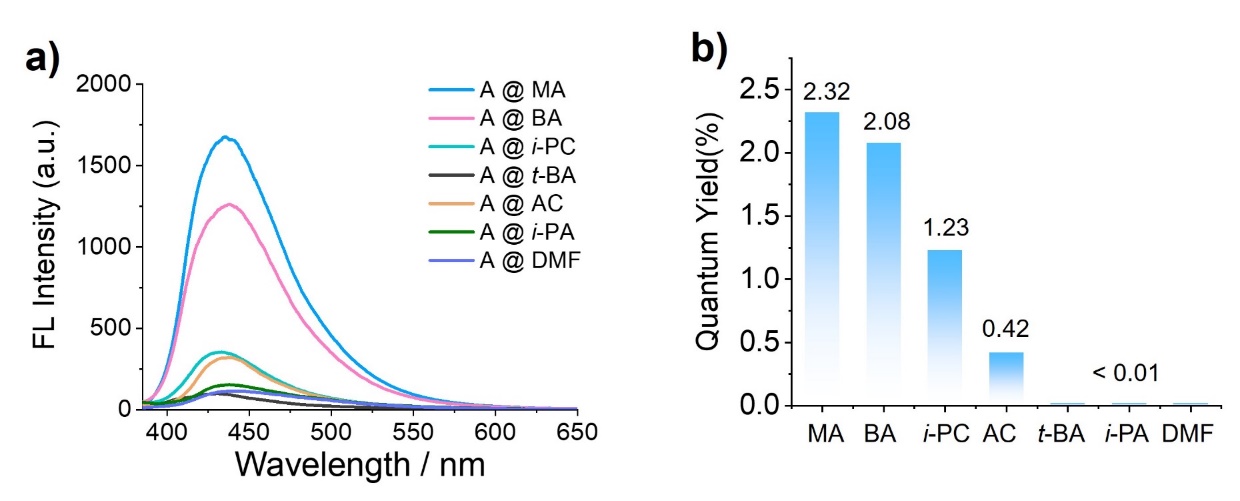


**Figure S4.** (a) Fluorescence spectra of A-MOF@guests, *λ*_ex_ = 360 nm. (b) The quantum yield of A-MOF@guests.


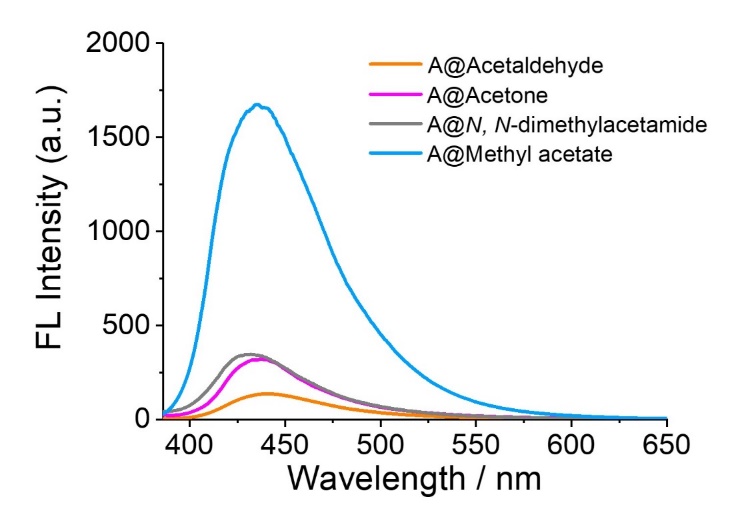


**Figure S5.** Fluorescence spectra of A-MOF@guests, *λ*_ex_ = 360 nm.

We also encapsulated the above four solvents molecules in A-MOF and tested their fluorescence spectra. The test results showed that the fluorescence intensity of A-MOF@Acetaldehyde was lower than that of acetone, while the fluorescence intensity of *N, N*-dimethylacetamide was similar to that of acetone, but weaker than that of methyl acetate. Based on the electron donating ability of the guest molecules encapsulated in A-MOF, acetaldehyde, acetone, methyl acetate, and *N, N*-dimethylacetamide should exhibit gradually increasing fluorescence. However, experimental results show that *N, N*-dimethylacetamide is weaker than methyl acetate, possibly due to steric hindrance effects.


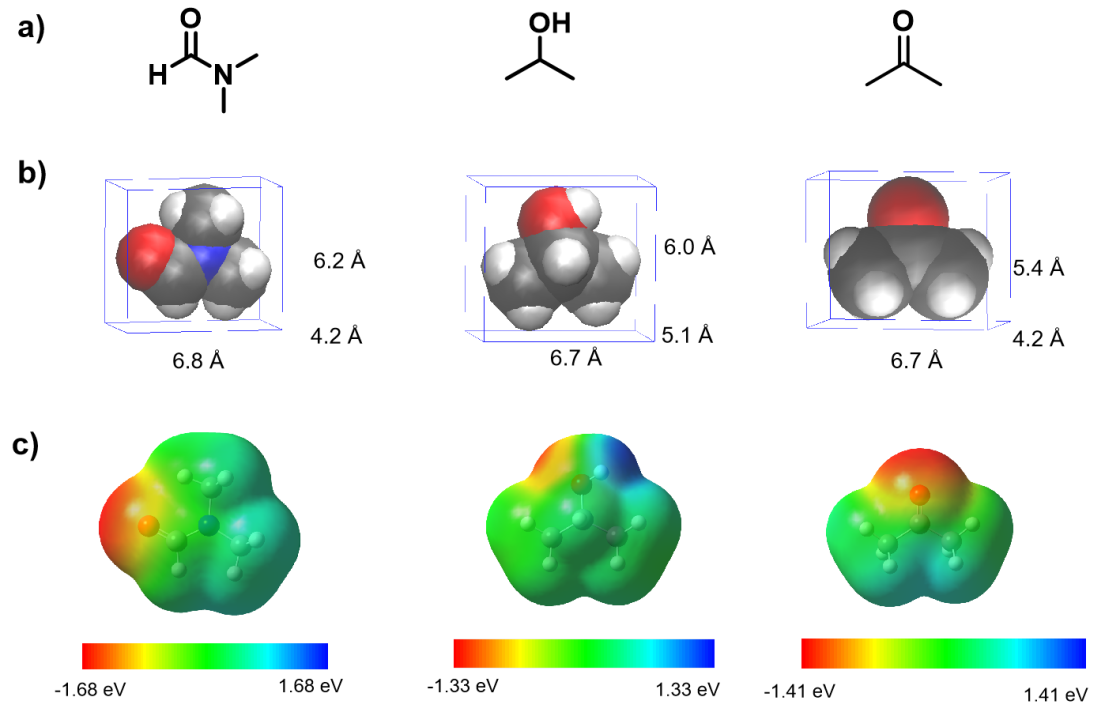


**Figure S6.** (a) Molecular structural formula. (b) Optimization of molecular dimensions. (c) Molecular electrostatic potential. Calculated by Density Functional Theory (DFT), Gaussian 16, b3lyp/6-311G(d,p) and Multiwfn 3.8 (dev).


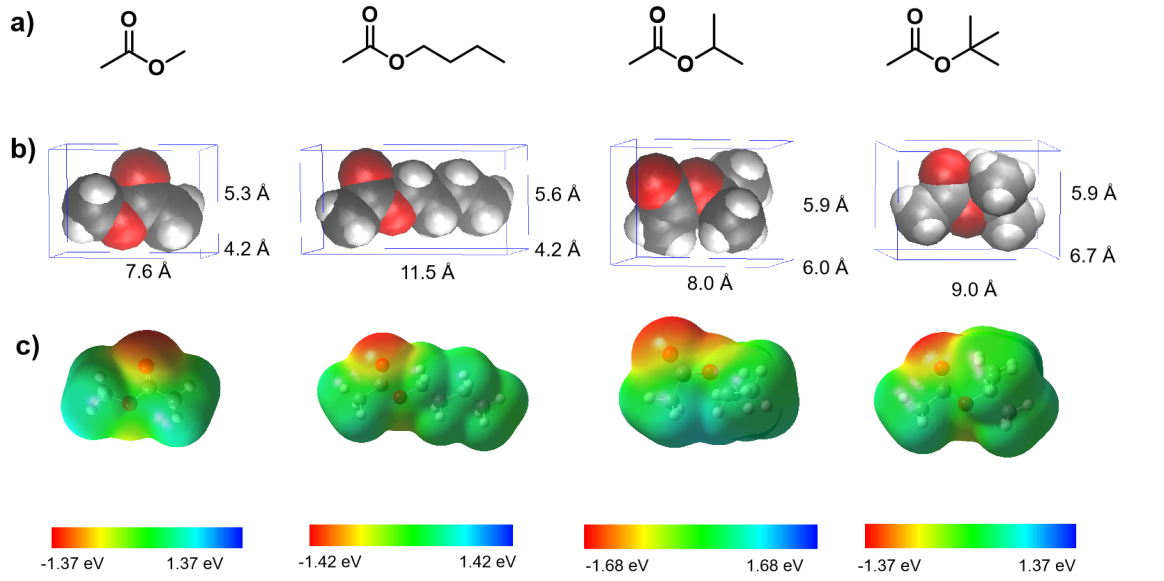


**Figure S7.** (a) Ester molecular formula. (b) Optimization of molecular dimensions. (c) Molecular electrostatic potential. Calculated by Density Functional Theory (DFT), Gaussian 16, b3lyp/6-311G(d,p) and Multiwfn 3.8 (dev).


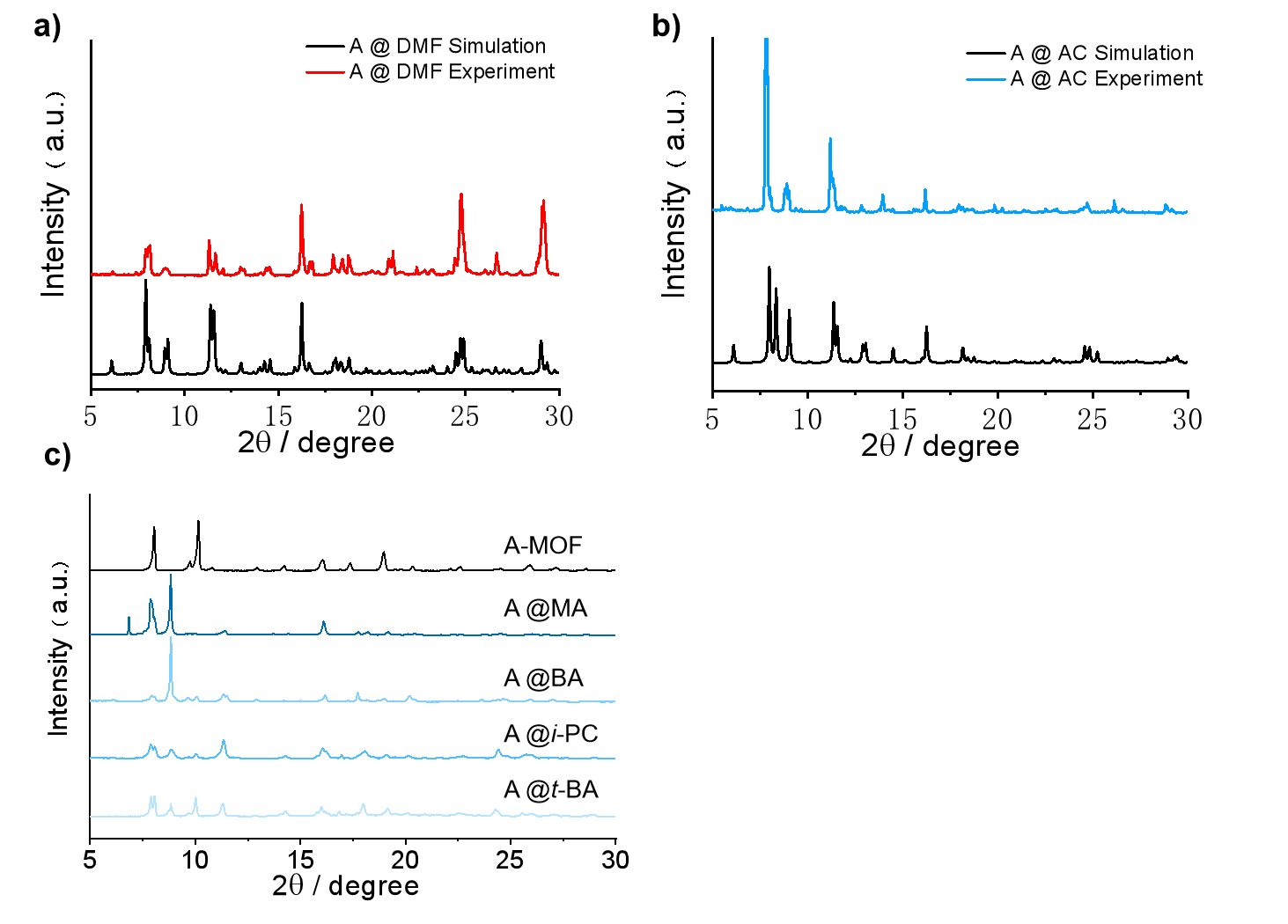


**Figure S8.** XRD of A-MOF@DMF (a); A-MOF@Acetone (b); A-MOF@guest (c) (A-MOF solventless, methyl acetate, n-butyl acetate, isopropyl acetate, tert-butyl acetate).


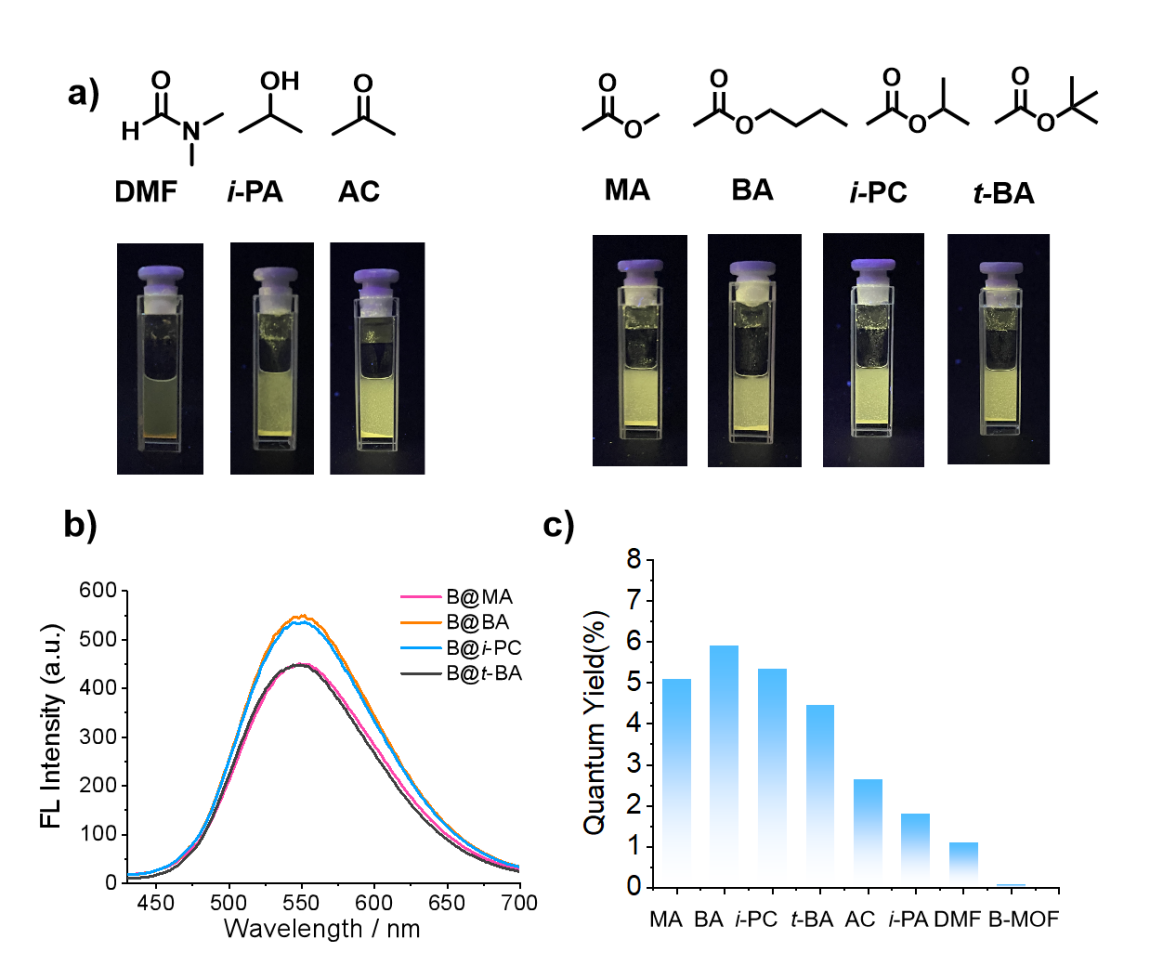


**Figure S9.** Fluorescence images of B-MOF with encapsulated solvent guest molecules: (a) lp-π interaction induced fluorescence "turn on" and size and steric effects. Fluorescence spectra of B-MOF with encapsulated methyl acetate, n-butyl acetate, isopropyl acetate, *t*-butyl acetate (b), *λ*_ex_ = 360 nm. (c) The quantum yield of B-MOF@guest.


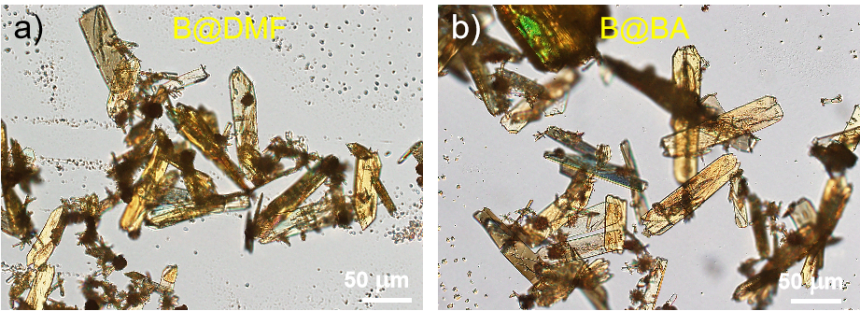


**Figure S10.** Fluorescence microscopy images: in the natural light (a) B-MOF@DMF and (b) B-MOF@BA.


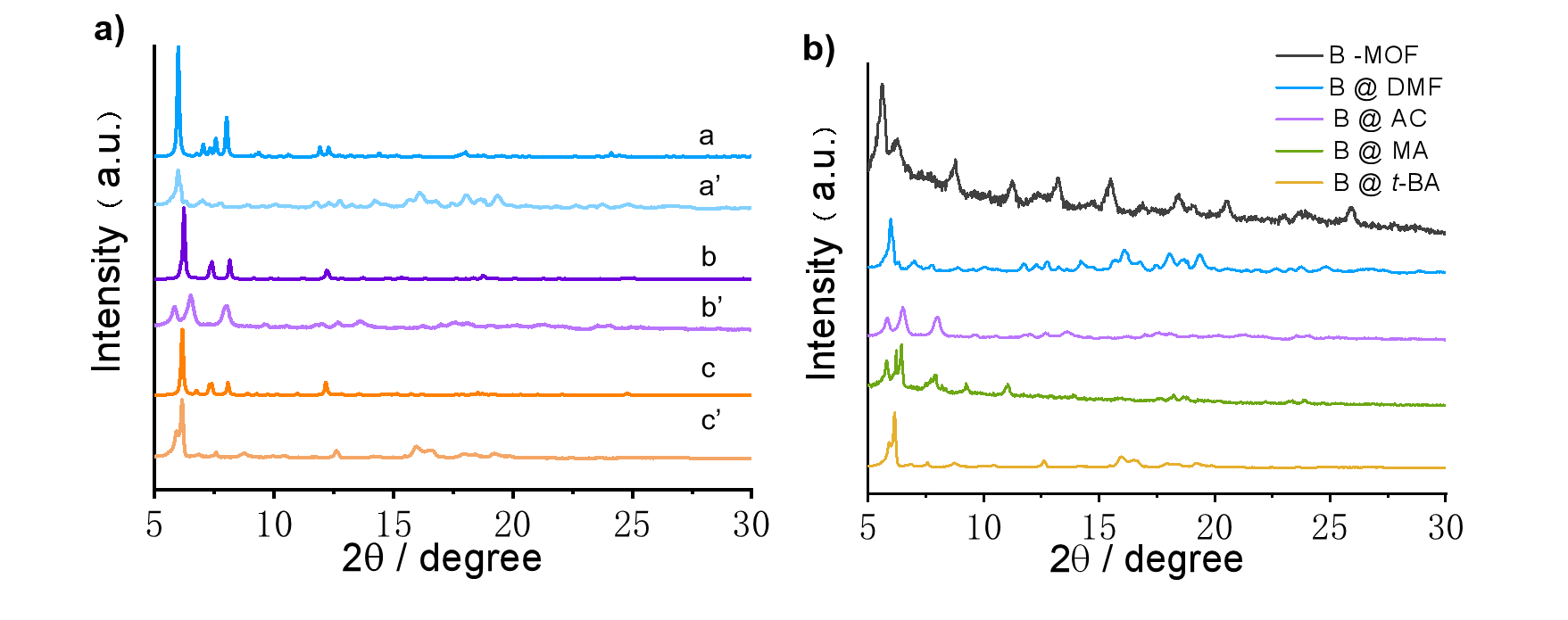


**Figure S11.** (a) XRD of B-MOF@DMF (a, a’), B-MOF@Acetone (b, b’), B-MOF@*t*-BA (c, c’); (a-c) Simulated PXRD obtained from single crystal, (a’-c’) Experimental measurement of PXRD. (b) PXRD of B-MOF@guest (guest: DMF, acetone, methyl acetate, tert-butyl acetate).


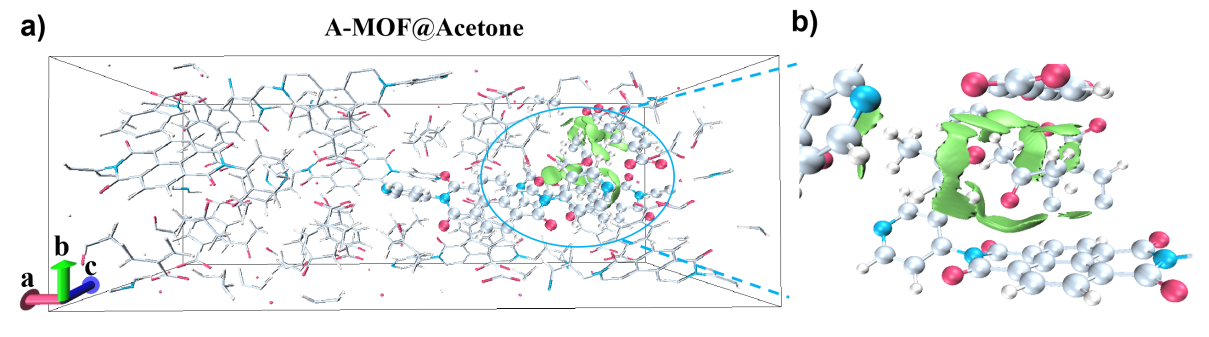


**Figure S12.** IGMH analysis of optimized structure of A-MOF@Acetone. Green isosurfaces showing the weak noncovalent interactions. (a) The image of full crystal cell. (b) Magnified image of intermolecular forces between the acetone molecule and surrounding molecules on the right side of the unit cell.


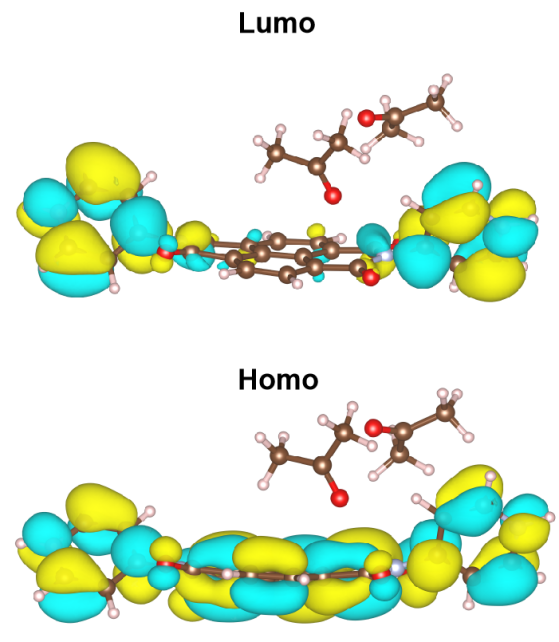


**Figure S13.** The HOMO (Highest Occupied Molecular Orbital) and LUMO (Lowest Unoccupied Molecular Orbital) energy levels of NDIPy and acetone molecules.

4. Adaptive chirality in achiral nanoconfined MOFs


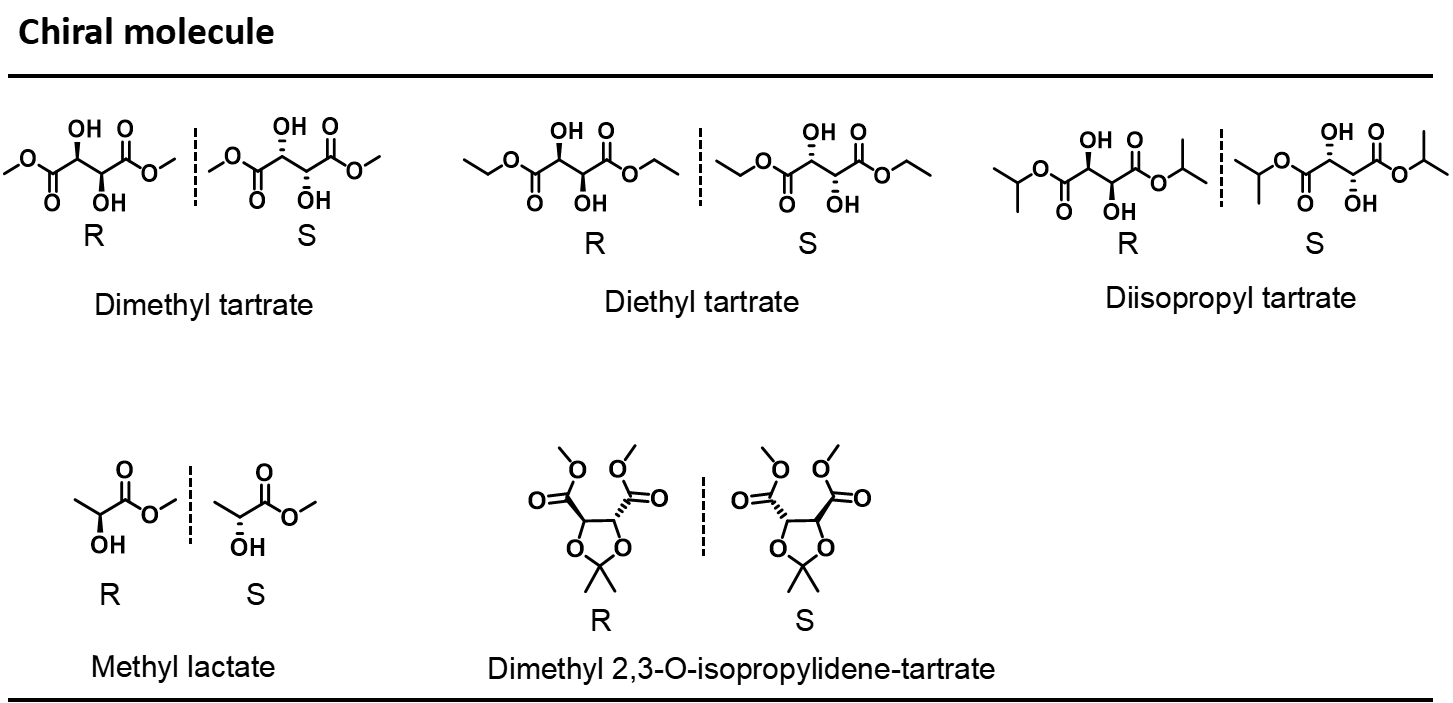


**Figure S14.** Chiral molecular structure formula.


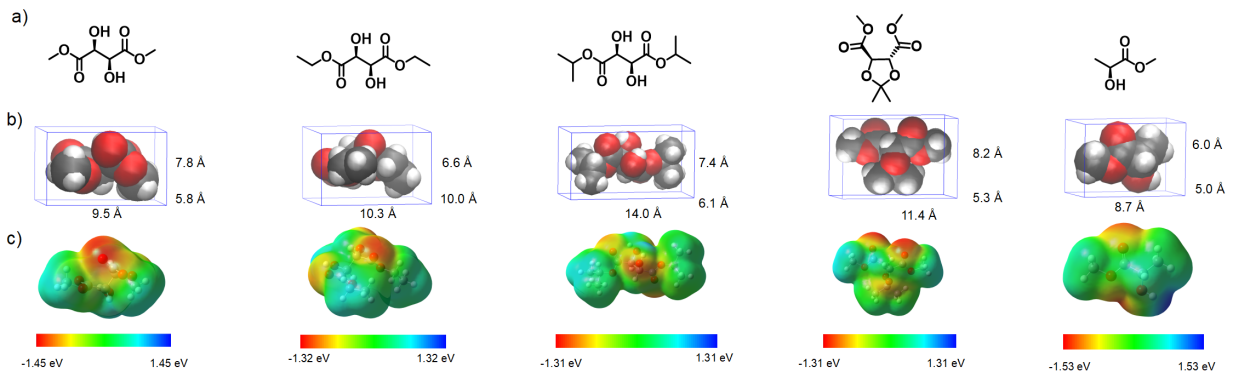


**Figure S15.** (a) Chiral molecular structure formula, (b) Optimization of molecular dimensions, (c) Molecular electrostatic potential. Calculated by Density Functional Theory (DFT), Gaussian 16, b3lyp/6-311G(d,p) and Multiwfn 3.8 (dev).


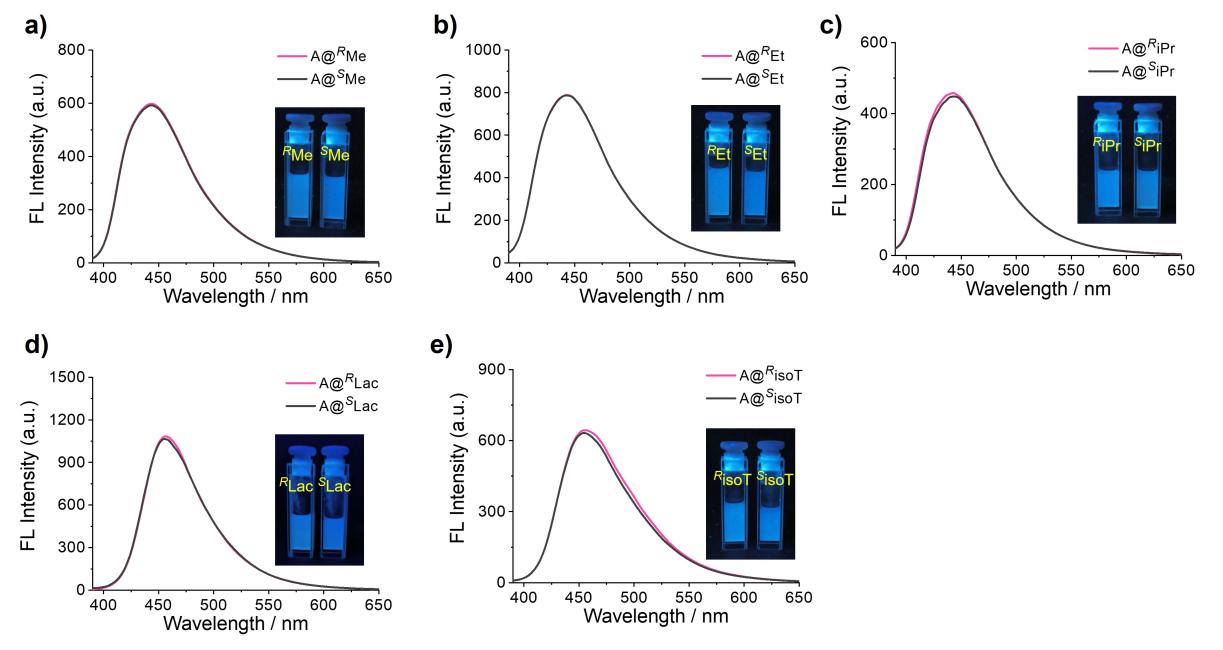


**Figure S16.** Fluorescence spectra of A-MOF@chiral guests, *λ*_ex_ = 360 nm. The inset fluorescence image depicts chiral guest molecules encapsulated within A-MOF.


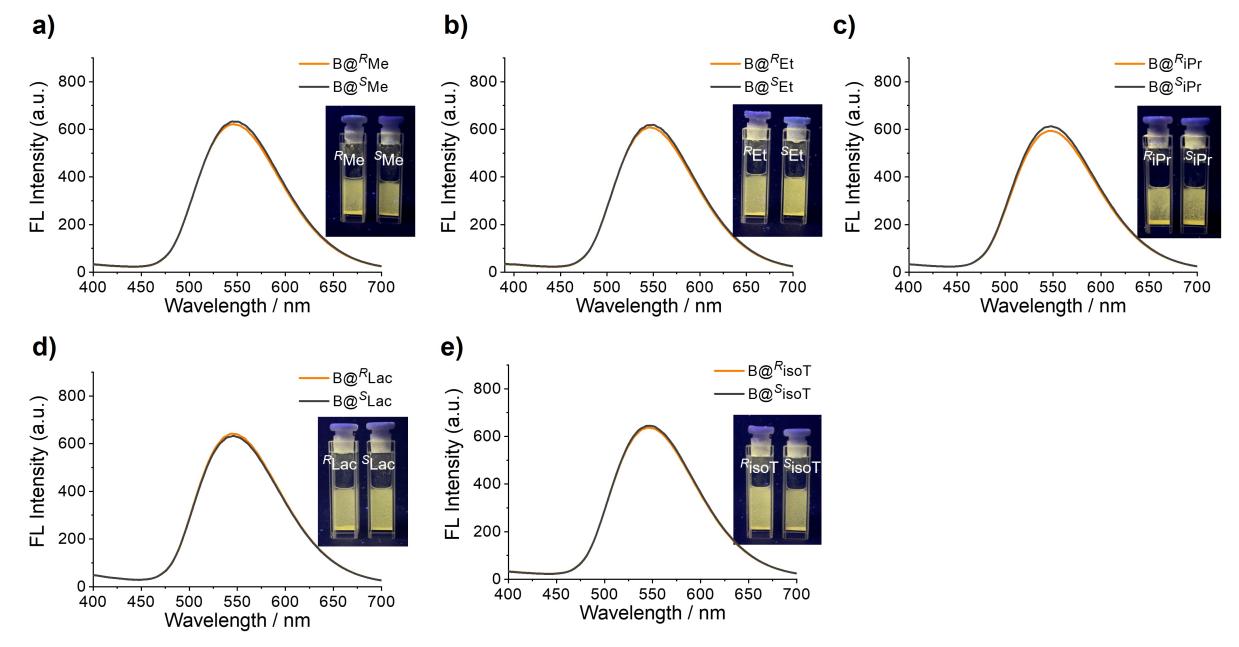


**Figure S17.** Fluorescence spectra of B-MOF@chiral guests, *λ*_ex_ = 360 nm. The inset fluorescence image depicts chiral guest molecules encapsulated within B-MOF.


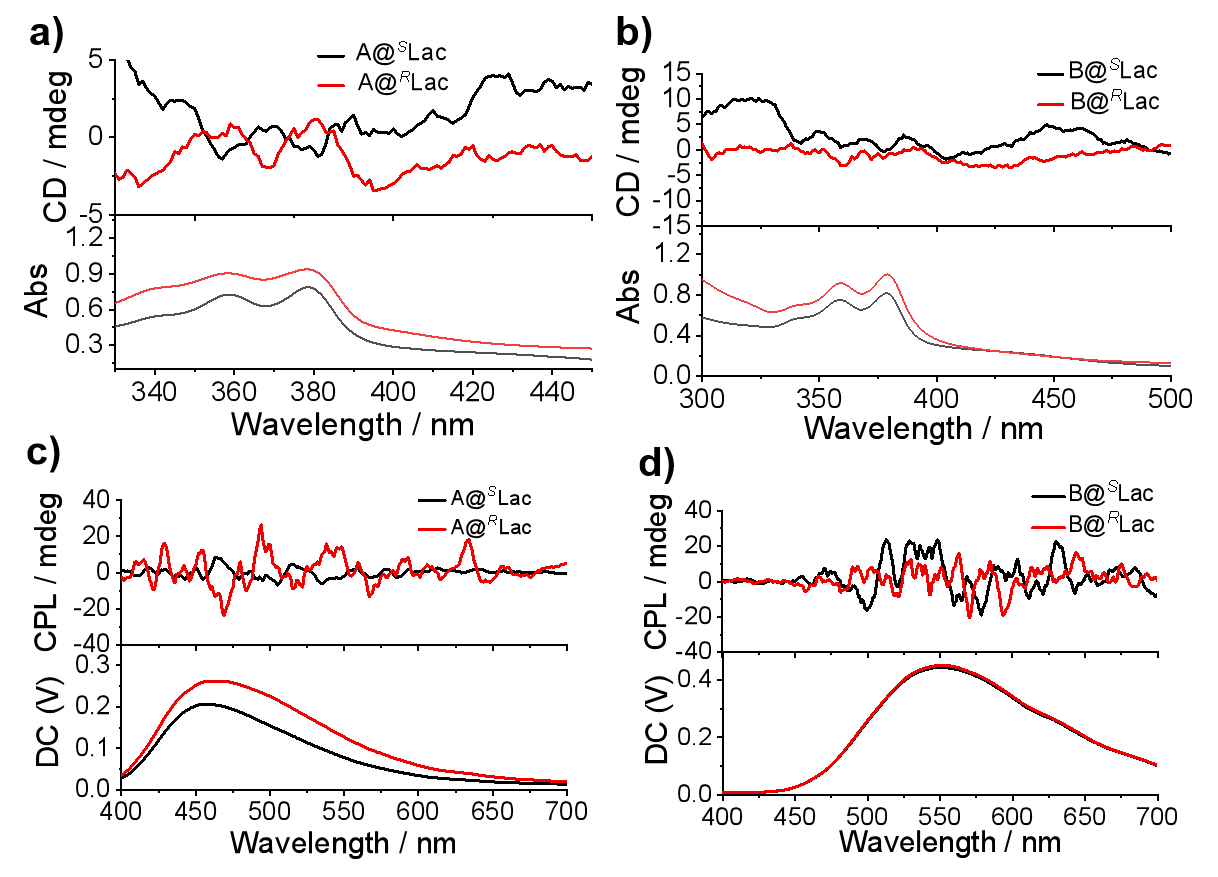


**Figure S18.** Chiroptical spectra of A-MOF@*^R/S^*Lac and B-MOF@*^R/S^*Lac. (a, b) CD spectra; (c, d) CPL spectra. *λ*_ex_ = 360 nm for CPL measurements.


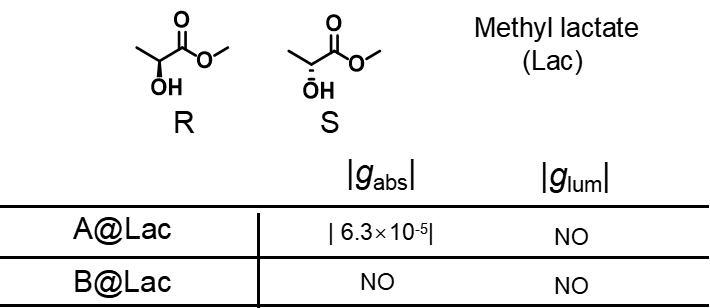


**Table S1.** Summary of chiroptical properties (*g*_abs_ and *g*_lum_) of A-MOF@*^R/S^*Lac and B-MOF@*^R/S^*Lac.


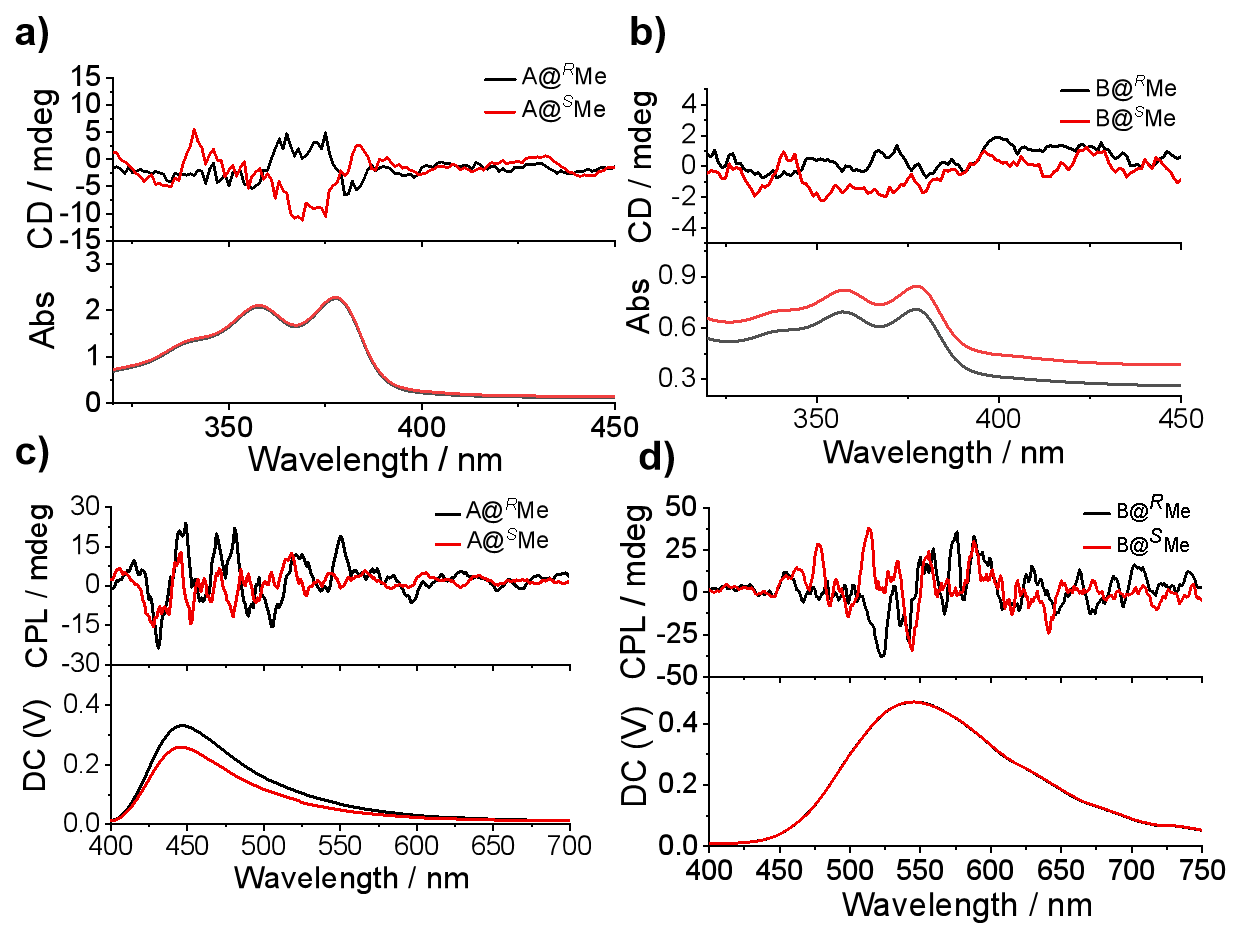


**Figure S19.** Chiroptical spectra of A-MOF@*^R/S^*Me and B-MOF@*^R/S^*Me. (a, b) CD spectra; (c, d) CPL spectra. *λ*_ex_ = 360 nm for CPL measurements.


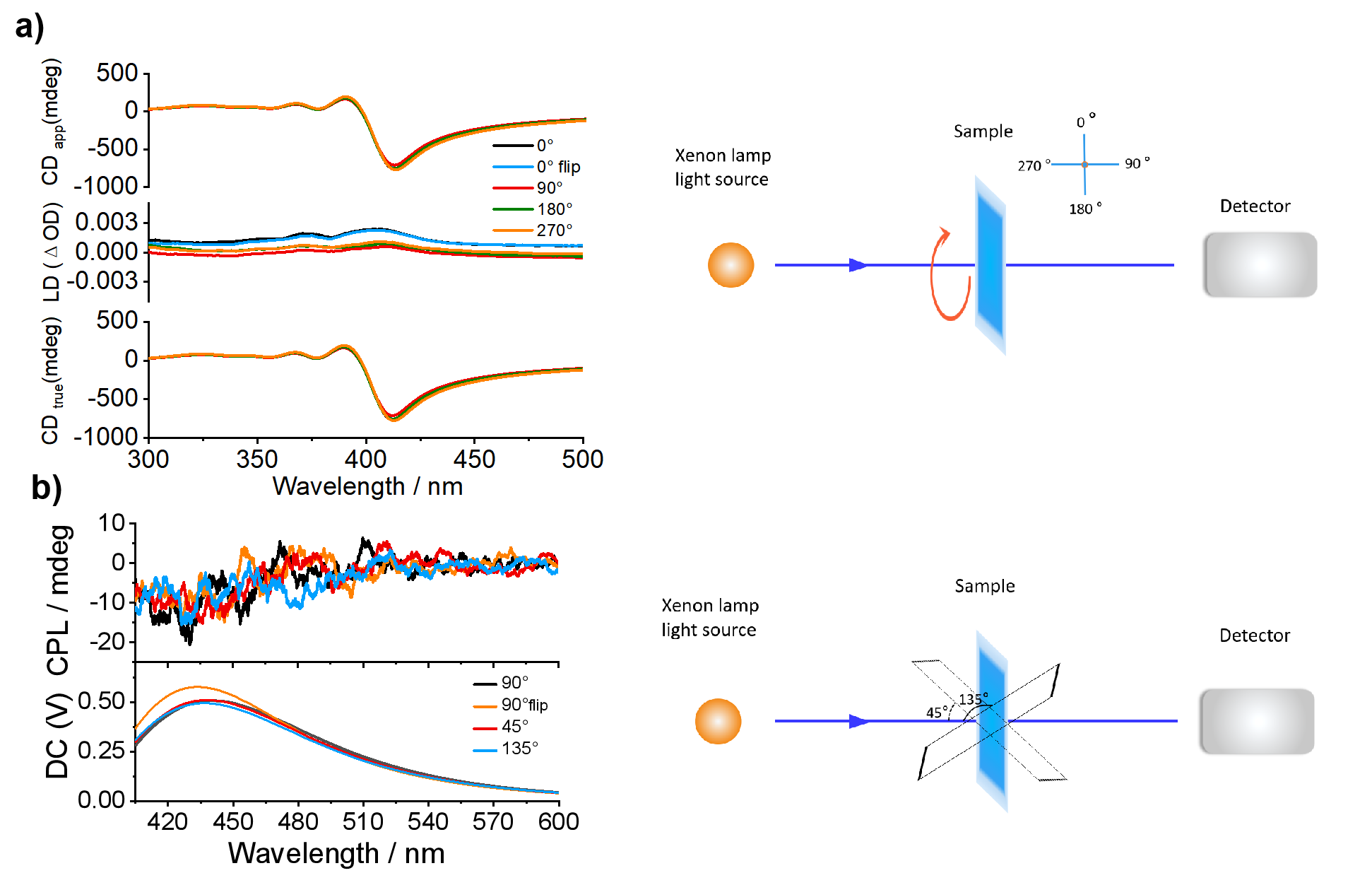


**Figure S20.** Chiroptical spectra of A-MOF@*^R^*Et. (a) CD spectra; (b) CPL spectra. *λ*_ex_ = 360 nm for CPL measurements. According to the approximation equation CD_true_ = CD_app_ - 0.02×LD, the contributions of LD to the true CD signals (CD_true_) are explored in the A-MOF@*^R^*Et by rotating and flipping the cuvette at 90º intervals around the direction of incident light propagation, where CD_app_ denotes the apparent CD signals captured from the CD photospectrometer. The CPL spectra of A-MOF@*^R^*Et are investigated by altering the angle of the sample and flipping it along the direction of incident light propagation, eliminated the phenomenon of linearly polarized birefringence.


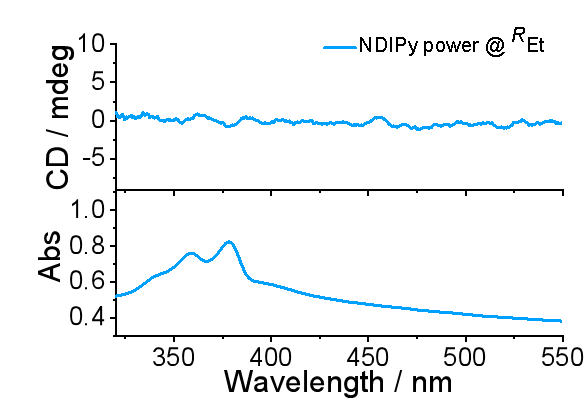


**Figure S21.** CD spectra of NDIPy power@*^R^*Et.


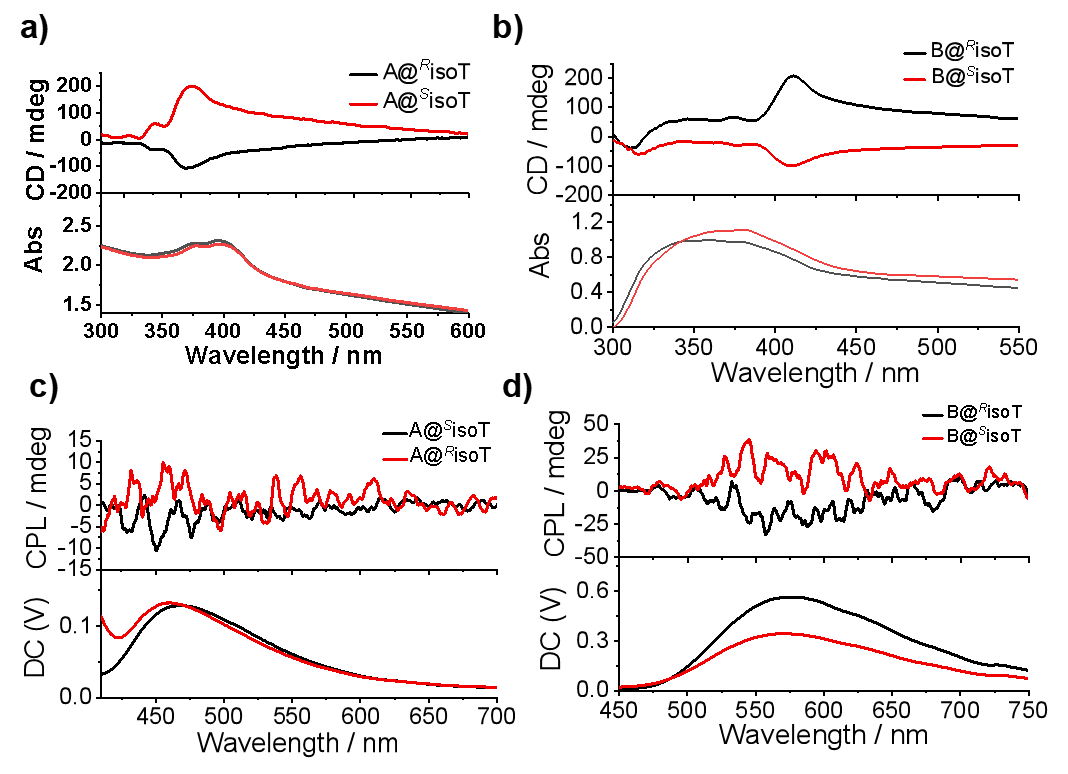


**Figure S22.** Chiroptical spectra of A-MOF@*^R/S^*isoT and B-MOF@*^R/S^*isoT. (a, b) CD spectra; (c, b) CPL spectra. *λ*_ex_ = 360 nm for CPL measurements.


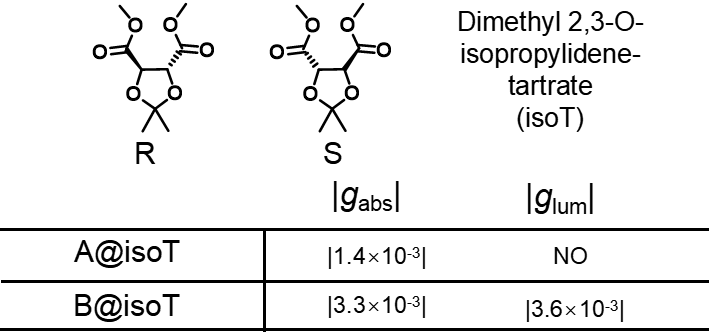


**Table S2.** Summary of chiroptical properties (*g*_abs_ and *g*_lum_) of A-MOF@*^R/S^*isoT and B-MOF@*^R/S^*isoT.


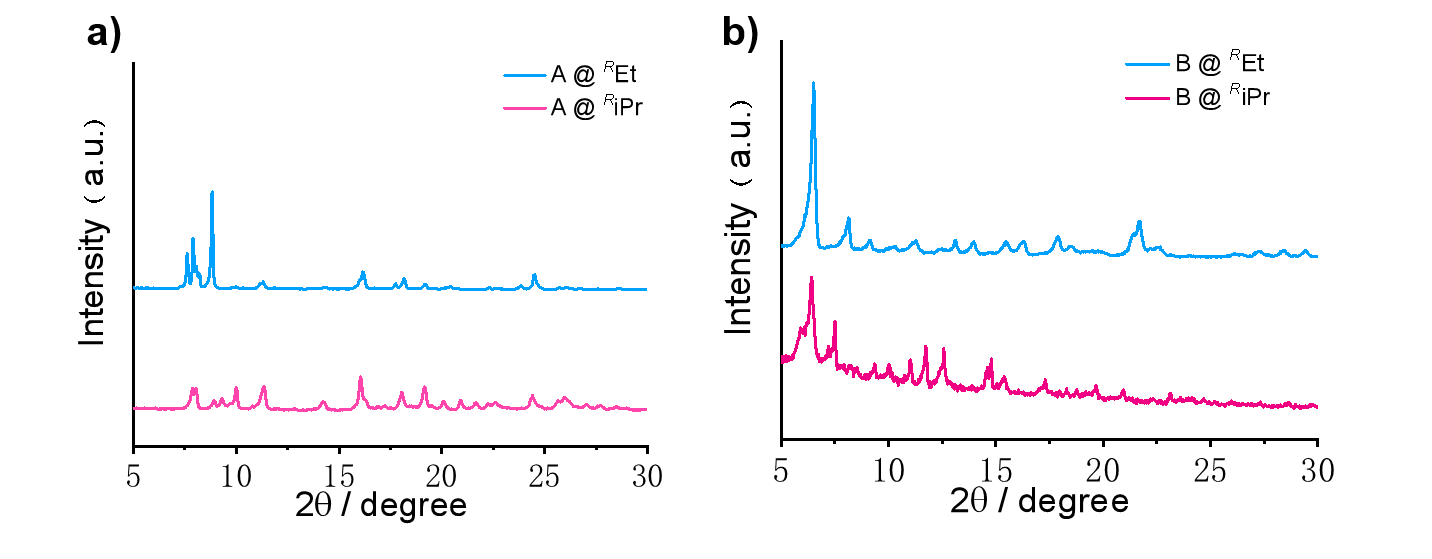


**Figure S23.** XRD of A-MOF@*^R/S^*Et and A-MOF@*^R/S^*iPr (a); B-MOF@*^R/S^*Et and B-MOF@*^R/S^*iPr (b).


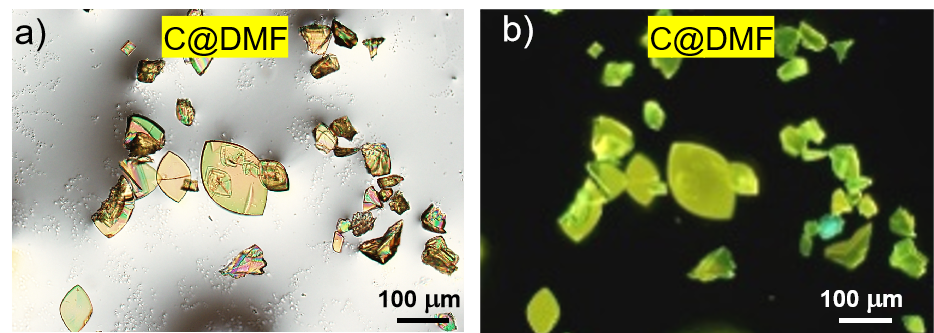


**Figure S24.** Fluorescence microscopy images of C-MOF@DMF: (a) in the natural light, (b) excited by DAPI (361-389 nm).


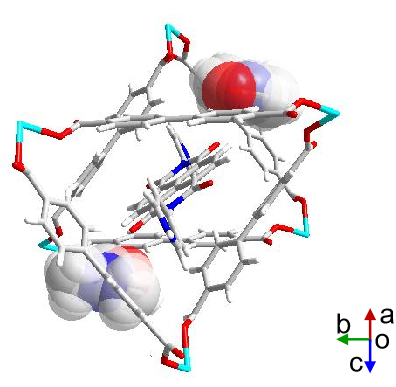


**Figure S25.** The crystal structure of C-MOF@DMF (CCDC: 2340871).


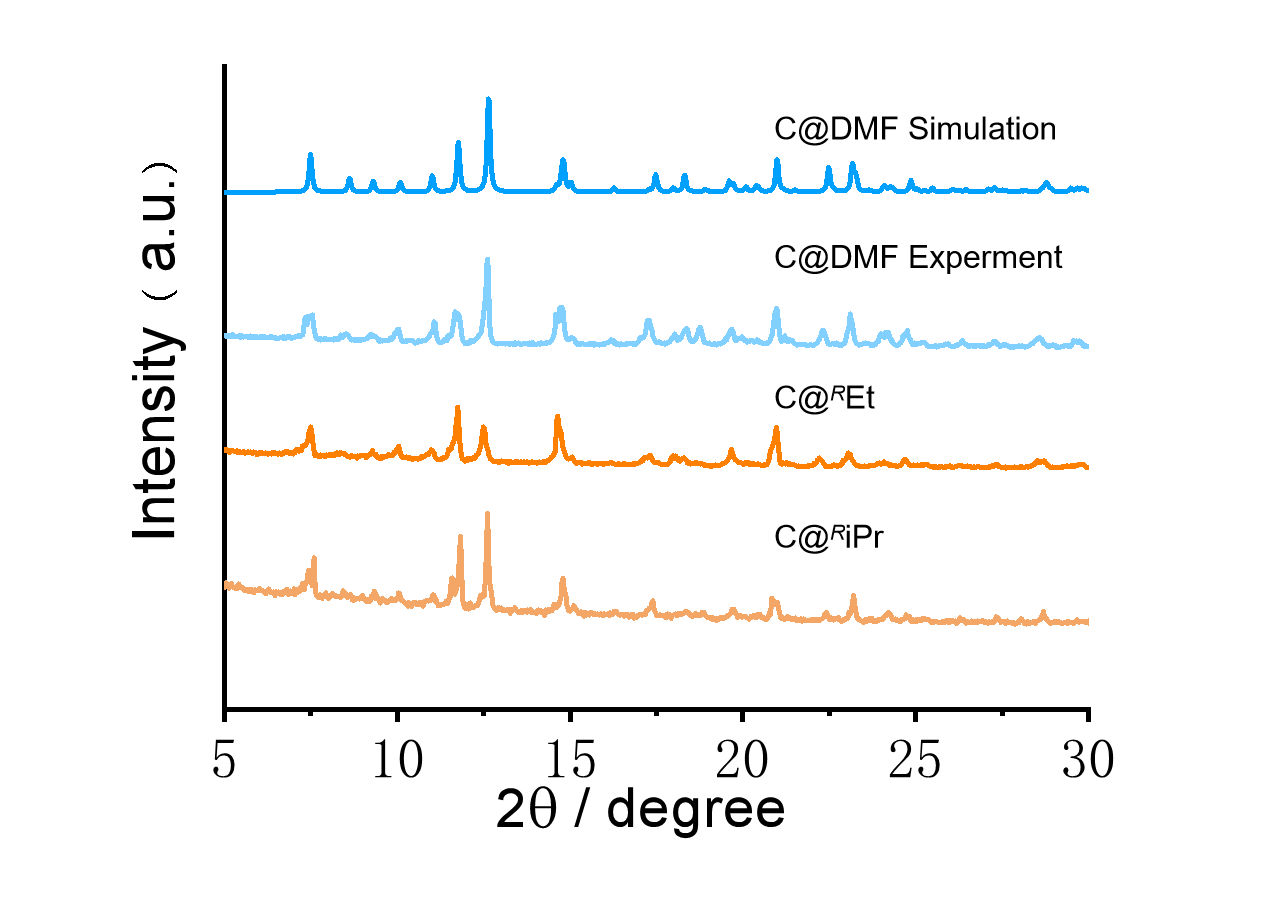


**Figure S26.** XRD of C-MOF@DMF、C-MOF@*^R^*Et and C-MOF@*^R^*iPr.


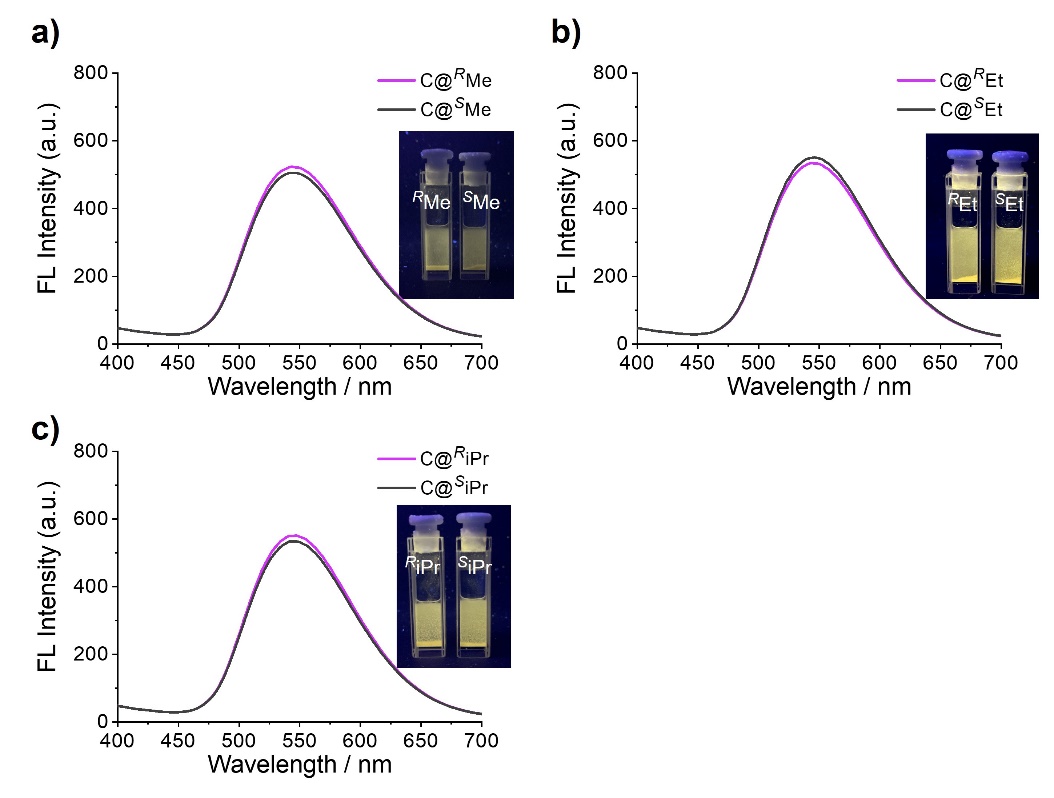


**Figure S27.** Fluorescence spectra of C-MOF@chiral guests, *λ*_ex_ = 360 nm. The inset fluorescence image depicts chiral guest molecules encapsulated within C-MOF.


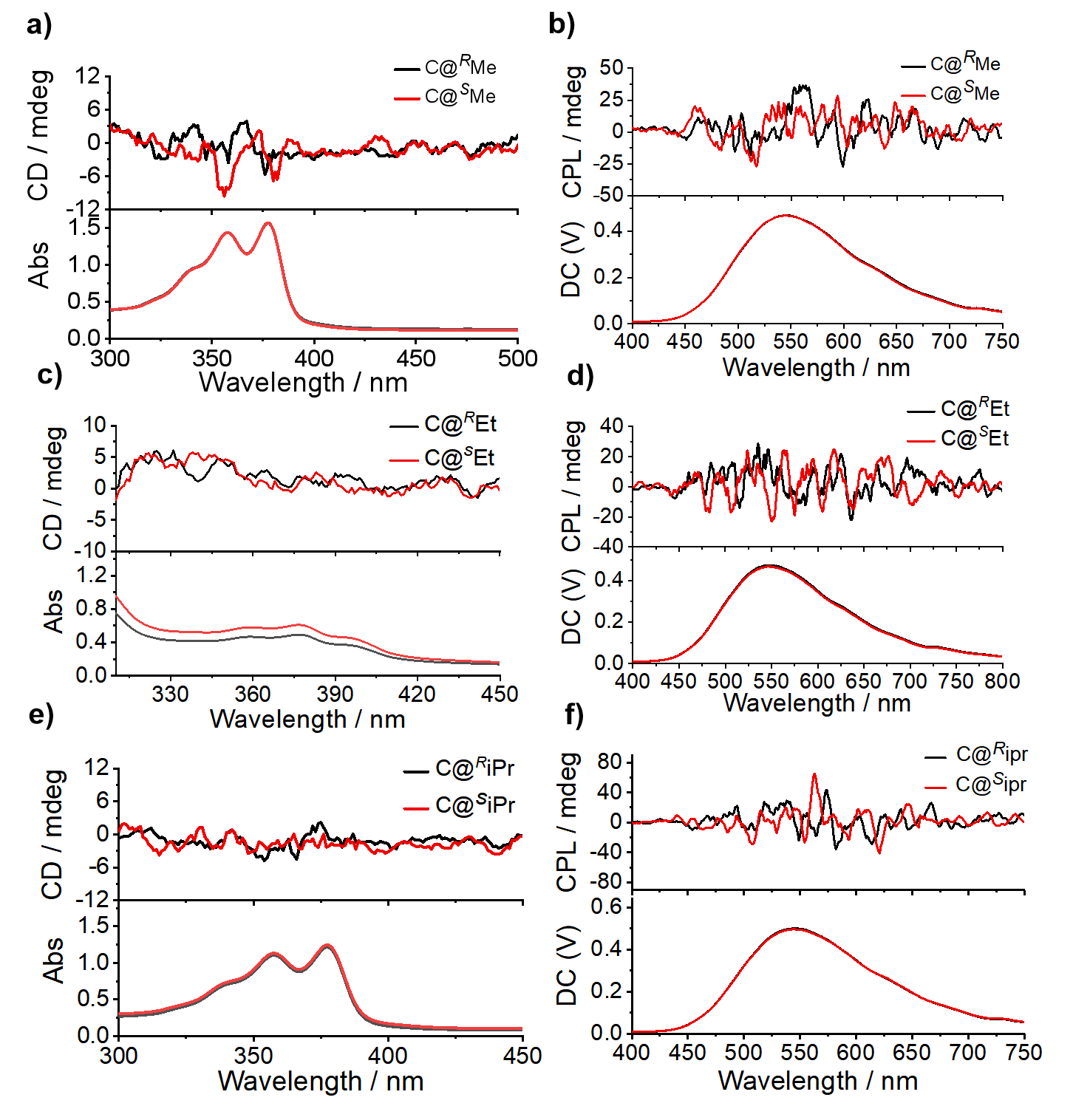


**Figure S28.** Chiroptical spectra of C-MOF@*^R/S^*Me, C-MOF@*^R/S^*Et and C-MOF@*^R/S^*iPr. (a, c, e) CD spectra; (b, d, f) CPL spectra. *λ*_ex_ = 360 nm for CPL measurements.

5. Single crystal data

**Table S3**. Crystal data and structure refinement for A-MOF@DMF.


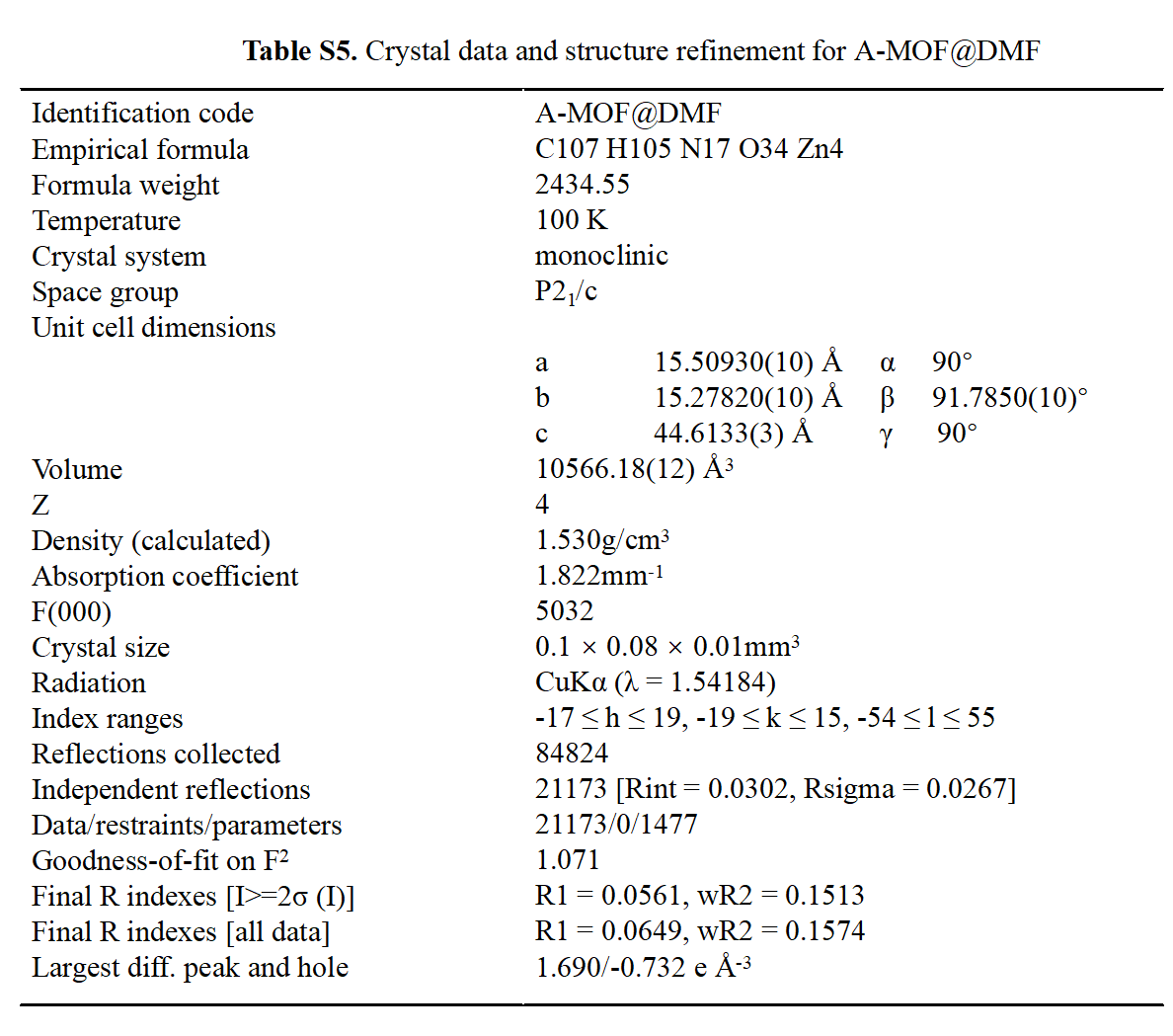


**Table S4**. Crystal data and structure refinement for A-MOF@Acetone.


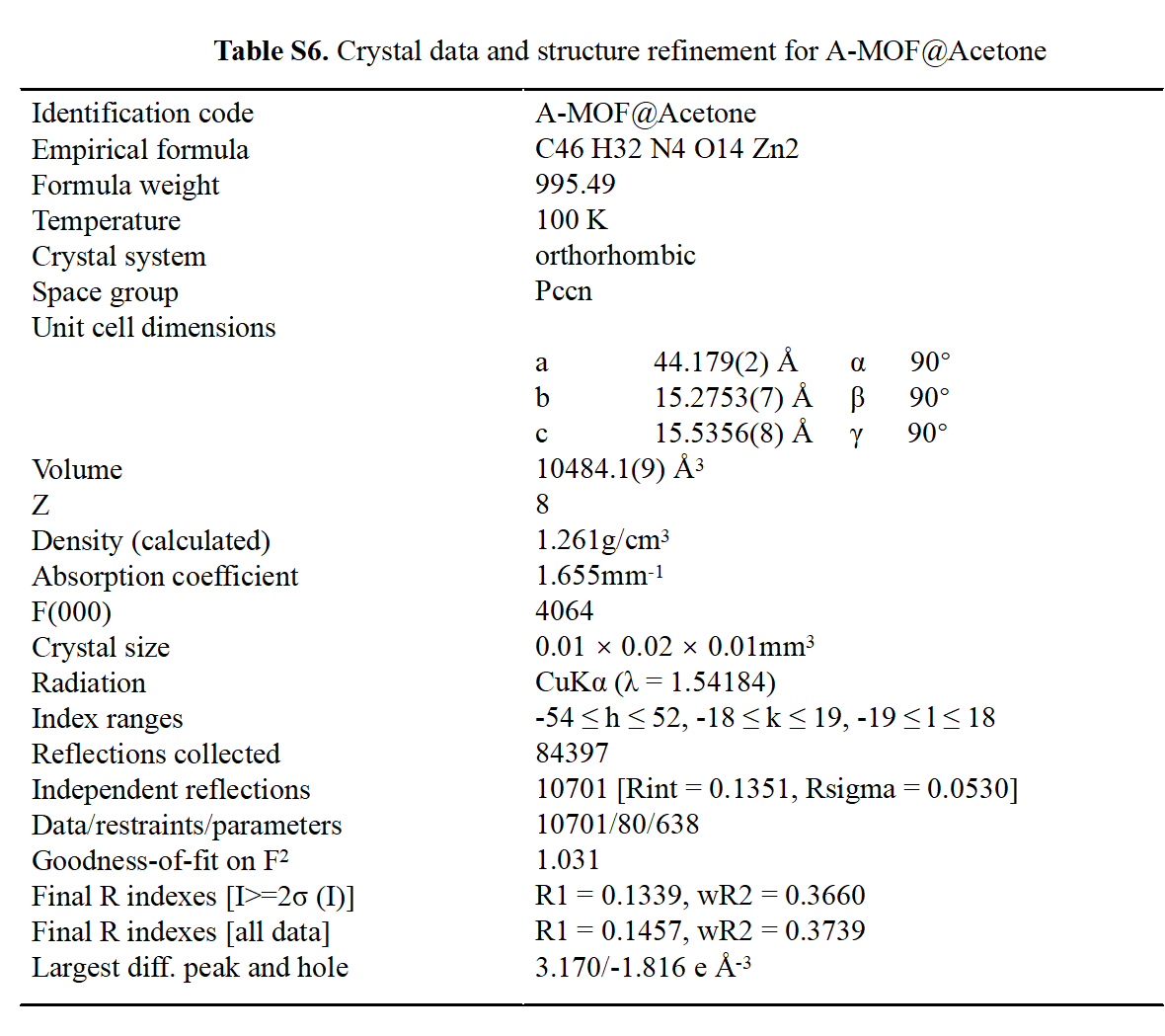


Table S5. Crystal data and structure refinement for B-MOF@DMF.


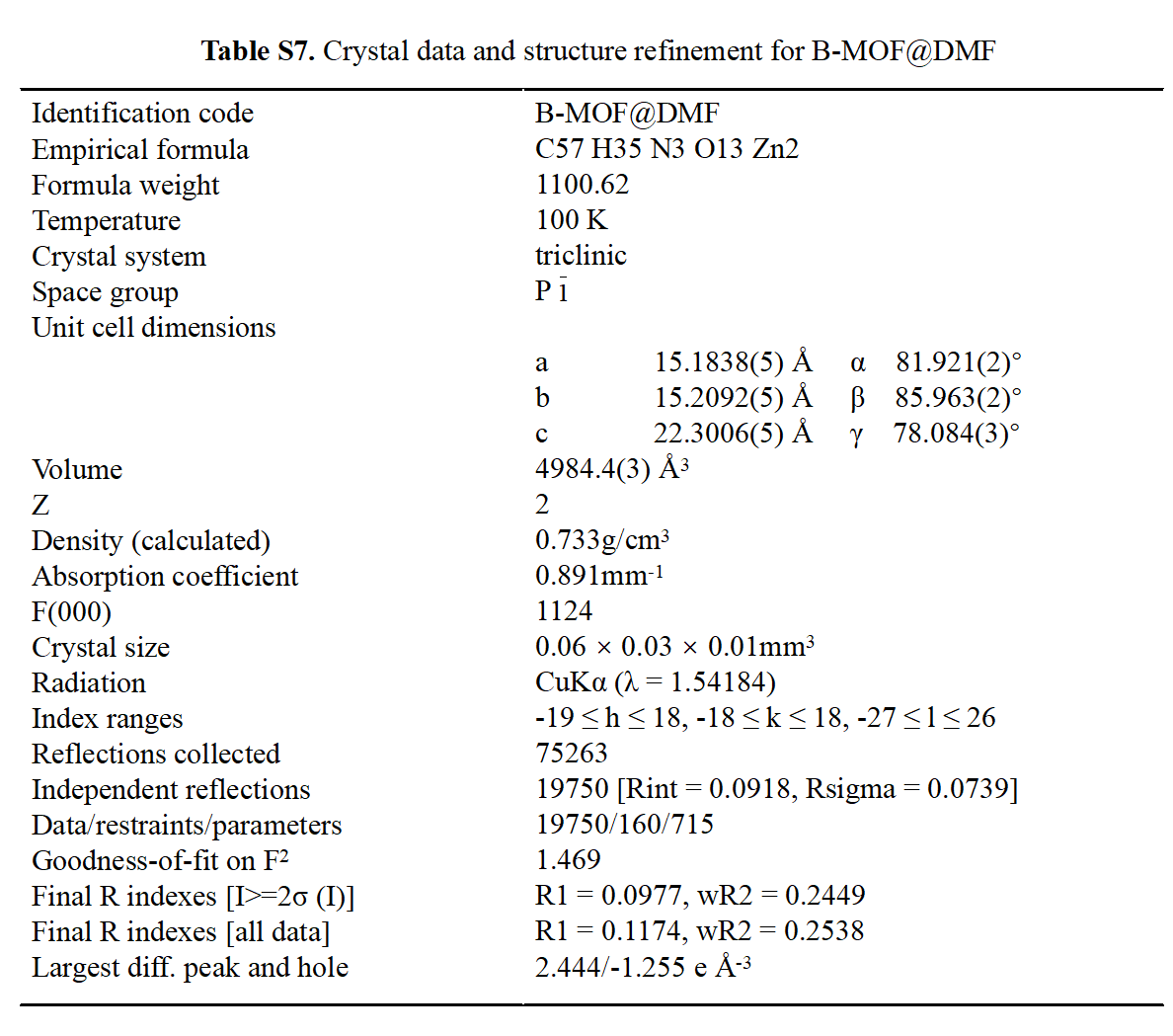


Table S6. Crystal data and structure refinement for B-MOF@Tert-buty acetate.


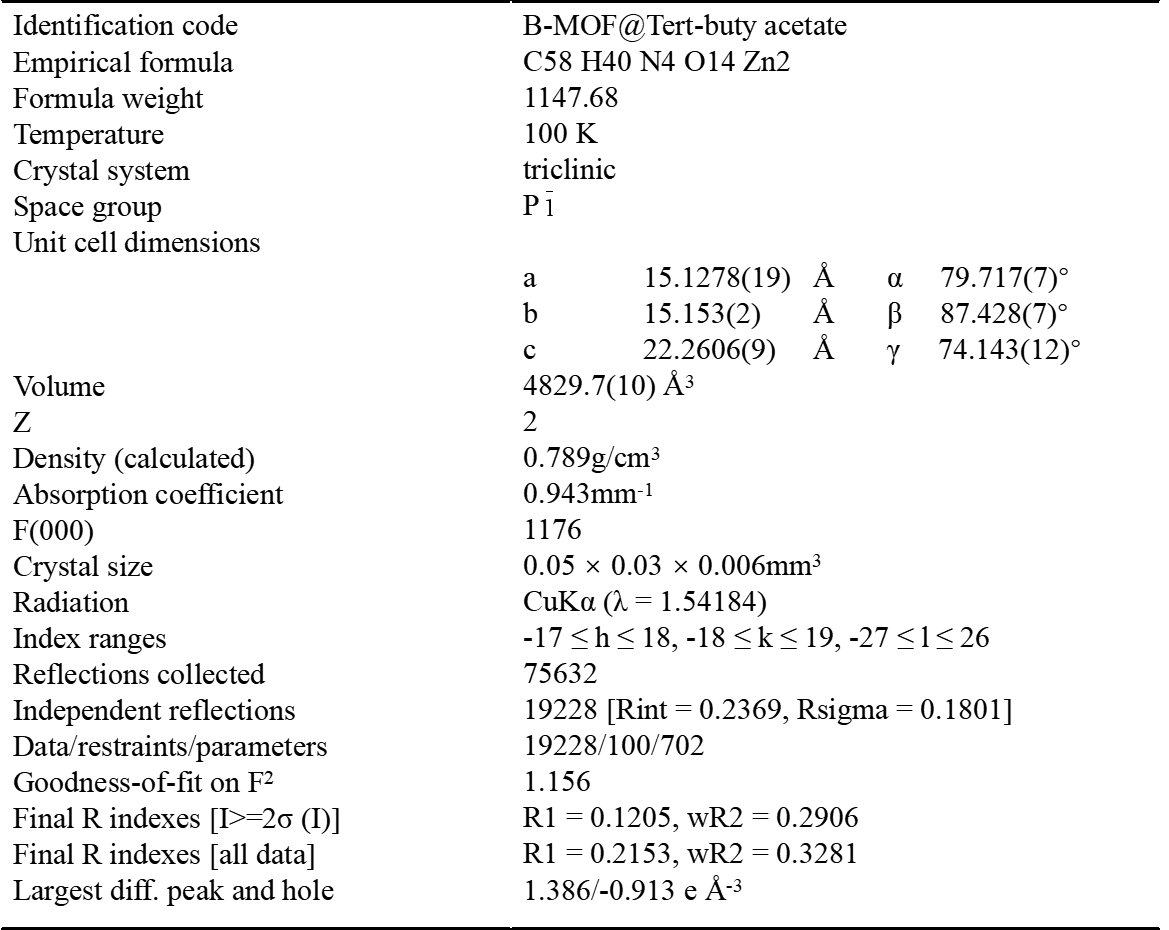


Table S7. Crystal data and structure refinement for B-MOF@Acetone.


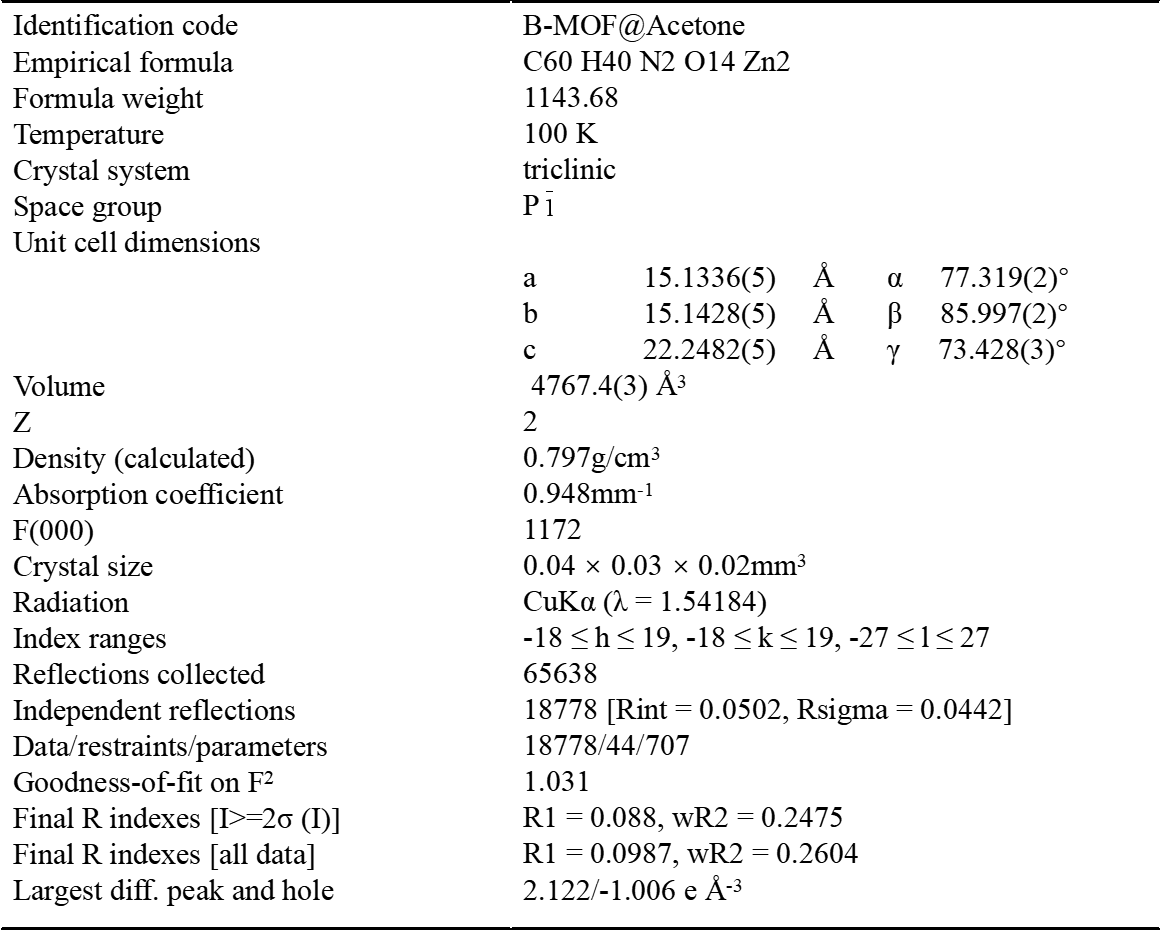


Table S8. Crystal data and structure refinement for C-MOF@DMF.


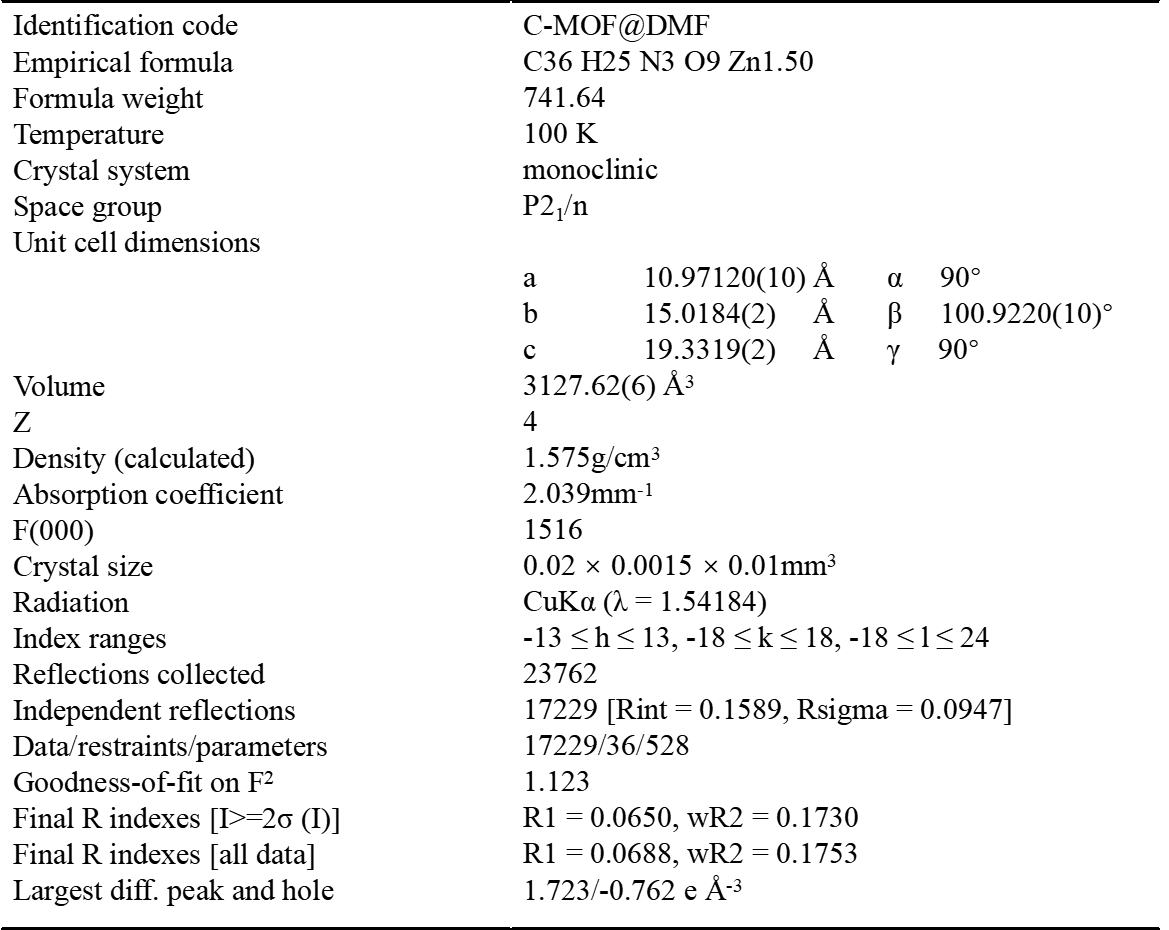


# 6. References

1. Frisch, M. J.; Trucks, G. W.; Schlegel, H. B.; Scuseria, G. E.; Robb, M. A.; Cheeseman, J. R.; Scalmani, G.; Barone, V.; Petersson, G. A.; Nakatsuji, H.; Li, X.; Caricato, M.; Marenich, A. V.; Bloino, J.; Janesko, B. G.; Gomperts, R.; Mennucci, B.; Hratchian, H. P.; Ortiz, J. V.; Izmaylov, A. F.; Sonnenberg, J. L.; Williams; Ding, F.; Lipparini, F.; Egidi, F.; Goings, J.; Peng, B.; Petrone, A.; Henderson, T.; Ranasinghe, D.; Zakrzewski, V. G.; Gao, J.; Rega, N.; Zheng, G.; Liang, W.; Hada, M.; Ehara, M.; Toyota, K.; Fukuda, R.; Hasegawa, J.; Ishida, M.; Nakajima, T.; Honda, Y.; Kitao, O.; Nakai, H.; Vreven, T.; Throssell, K.; Montgomery Jr., J. A.; Peralta, J. E.; Ogliaro, F.; Bearpark, M. J.; Heyd, J. J.; Brothers, E. N.; Kudin, K. N.; Staroverov, V. N.; Keith, T. A.; Kobayashi, R.; Normand, J.; Raghavachari, K.; Rendell, A. P.; Burant, J. C.; Iyengar, S. S.; Tomasi, J.; Cossi, M.; Millam, J. M.; Klene, M.; Adamo, C.; Cammi, R.; Ochterski, J. W.; Martin, R. L.; Morokuma, K.; Farkas, O.; Foresman, J. B.; Fox, D. J., Gaussian 16, Revision A.03, Gaussian, Inc., Wallingford, CT, **2016**.

2. Stephens, P. J.; Devlin, F. J.; Chabalowski, C. F.; Frisch, M. J., Ab initio calculation of vibrational absorption and circular dichroism spectra using density functional force fields. *J. Phys. Chem.* **1994**, *98* (45), 11623-11627.

3. Grimme, S.; Ehrlich, S.; Goerigk, L., Effect of the damping function in dispersion corrected density functional theory. *J. Comput. Chem.* **2011**, *32* (7), 1456-1465.

4. Kühne, T. D.; Iannuzzi, M.; Del Ben, M.; Rybkin, V. V.; Seewald, P.; Stein, F.; Laino, T.; Khaliullin, R. Z.; Schütt, O.; Schiffmann, F., CP2K: An electronic structure and molecular dynamics software package-Quickstep: Efficient and accurate electronic structure calculations. *J. Phys. Chem.* **2020**, *152* (19).

5. Adamo, C.; Barone, V., Toward reliable density functional methods without adjustable parameters: The PBE0 model. *J. Chem. Phys.* **1999,** 110 (13), 6158-6170.

6. Guidon, M.; Hutter, J.; VandeVondele, J., Auxiliary density matrix methods for Hartree−Fock exchange calculations. *J. Chem. Theory Comput.* **2010**, 6 (8), 2348-2364.

7. Lefebvre, C.; Rubez, G.; Khartabil, H.; Boisson, J.-C.; Contreras-García, J.; Hénon, E., Accurately extracting the signature of intermolecular interactions present in the NCI plot of the reduced density gradient versus electron density. *Phys. Chem. Chem. Phys.* **2017**, *19* (27), 17928-17936.

8. Lu, T.; Chen, F., Multiwfn: A multifunctional wavefunction analyzer. *J. Comput. Chem.* **2012**, *33* (5), 580-592.

9. Lu, T.; Chen, Q., Independent gradient model based on Hirshfeld partition: A new method for visual study of interactions in chemical systems. *J. Comput. Chem.* **2022**, *43* (8), 539-555.

10. Humphrey, W.; Dalke, A.; Schulten, K., VMD: visual molecular dynamics. *J. Mol. Graph.* **1996**, *14* (1), 33-38.

11. Momma, K.; Izumi, F., VESTA 3 for three-dimensional visualization of crystal, volumetric and morphology data. *J. Appl. Crystallogr.* **2011**, *44* (6), 1272-1276.
